# Gateways to the FANTOM5 promoter level mammalian expression atlas

**DOI:** 10.1186/s13059-014-0560-6

**Published:** 2015-01-05

**Authors:** Marina Lizio, Jayson Harshbarger, Hisashi Shimoji, Jessica Severin, Takeya Kasukawa, Serkan Sahin, Imad Abugessaisa, Shiro Fukuda, Fumi Hori, Sachi Ishikawa-Kato, Christopher J Mungall, Erik Arner, J Kenneth Baillie, Nicolas Bertin, Hidemasa Bono, Michiel de Hoon, Alexander D Diehl, Emmanuel Dimont, Tom C Freeman, Kaori Fujieda, Winston Hide, Rajaram Kaliyaperumal, Toshiaki Katayama, Timo Lassmann, Terrence F Meehan, Koro Nishikata, Hiromasa Ono, Michael Rehli, Albin Sandelin, Erik A Schultes, Peter AC ‘t Hoen, Zuotian Tatum, Mark Thompson, Tetsuro Toyoda, Derek W Wright, Carsten O Daub, Masayoshi Itoh, Piero Carninci, Yoshihide Hayashizaki, Alistair RR Forrest, Hideya Kawaji, the FANTOM consortium

**Affiliations:** Omics Science Center, RIKEN Yokohama Institute, 1-7-22 Suehiro-cho, Tsurumi-ku, Yokohama, Kanagawa 230-0045 Japan; Division of Genomic Technologies (DGT), RIKEN Center for Life Science Technologie, 1-7-22 Suehiro-cho, Tsurumi-ku, Yokohama, Kanagawa 230-0045 Japan; RIKEN Preventive Medicine and Diagnosis Innovation Program, 2-1 Hirosawa, Wako, Saitama 351-0198 Japan; Preventive medicine and applied genomics unit, RIKEN Advanced Center for Computing and Communication, 1-7-22 Suehiro-cho, Tsurumi-ku, Yokohama, Kanagawa 230-0045 Japan; Genomics Division, Lawrence Berkeley National Laboratory, 84R01, 1 Cyclotron Road, Berkeley, CA 94720 USA; Mouse Informatics, European Molecular Biology Laboratory, European Bioinformatics Institute, Wellcome Trust Genome Campus, Hinxton, Cambridge CB10 1SD UK; The Roslin Institute and Royal (Dick) School of Veterinary Studies, University of Edinburgh, Easter Bush, Edinburgh, Midlothian, EH25 9RG Scotland UK; Department of Human Genetics, BioSemantics Group, Leiden University Medical Center, Albinusdreef 2, Leiden, 2333 ZA Netherlands; Department of Internal Medicine III, University Hospital Regensburg, F.-J.-Strauss Allee 11, Regensburg, D-93042 Germany; Database Center for Life Science, Research Organization of Information and Systems, 1111 Yata, Mishima, 411-8540 Japan; Department of Biology & Biotech Research and Innovation Centre, Section for Computational and RNA Biology, Copenhagen University, Ole Maaloes Vej 5, Copenhagen, N DK2200 Denmark; Department of Biostatistics, Harvard School of Public Health, 655 Huntington Avenue, Boston, MA 02115 USA; Department of Neurology, University at Buffalo School of Medicine and Biomedical Sciences, 701 Ellicott Street, Buffalo, NY 14203 USA; BioSemantics Group, Leiden Institute of Advanced Computer Science, Leiden University, 111 Snellius, Niels Bohrweg 1, Leiden, 2333 CA Netherlands; Database Center for Life Science, Research Organization of Information and Systems, 178-4-4 Wakashiba, Kashiwa, Chiba 277-0081 Japan; Integrated Database Unit, RIKEN Advanced Center for Computing and Communication, 2-1 Hirosawa, Wako, Saitama 351-0198 Japan; Sheffield Institute for Translational Neuroscience, University of Sheffield, 385a Glossop Road, Sheffield, S10 2HQ UK; Telethon Kids Institute, The University of Western Australia, Perth, Western Australia 6008 Australia; Cancer Science Institute of Singapore, National University of Singapore, Singapore, 117599 Singapore

## Abstract

The FANTOM5 project investigates transcription initiation activities in more than 1,000 human and mouse primary cells, cell lines and tissues using CAGE. Based on manual curation of sample information and development of an ontology for sample classification, we assemble the resulting data into a centralized data resource (http://fantom.gsc.riken.jp/5/). This resource contains web-based tools and data-access points for the research community to search and extract data related to samples, genes, promoter activities, transcription factors and enhancers across the FANTOM5 atlas.

**Electronic supplementary material:**

The online version of this article (doi:10.1186/s13059-014-0560-6) contains supplementary material, which is available to authorized users.

## Introduction

One of the most comprehensive ways to study the molecular basis of cellular function is to quantify the presence of RNA molecules expressed by a given cell type. Over the years, the genomics field has collectively built up several gene expression repositories across biological states to facilitate exploration of biological systems. As for genome-wide surveys of encoded RNAs, a number of partial and full-length cDNA clone collections have been constructed and sequenced previously [1-6]. The resulting data were used for genome annotation, in particular to build gene models (NCBI RefSeq [4], Ensembl transcripts [7], Representative Transcript and Protein Sets (RTPS) [8]), and for exploration of active genes within specific biological contexts (NCBI UniGene [4], DigiNorthern [9], and cross-species analysis based on simplified ontologies [10]). However, the ability of these surveys to quantify RNA abundance was limited mainly due to sequencing performance. Another approach to assess gene expression is by hybridization to pre-designed probes (that is, microarrays) [11-13]. Thousands of studies have been published on gene expression profiles using microarrays (Gene Expression Omnibus [14], ArrayExpress [15], CIBEX [16]) and collections of curated data sets (GNF SymAtlas2 [17], EBI Gene expression atlas [18], BioGPS [19]) have become popular tools to survey gene expression levels. However, the coverage of identifiable RNA molecules and the accuracy of quantification are limited due to their probe design, which relies on existing knowledge of RNA species.

The recent development of next-generation sequencers enables us to obtain genome-wide RNA profiles comprehensively, quantitatively and without any pre-determination of what should be expressed using methods like cap analysis of gene expression (CAGE) [20] and RNA-seq [21]. In particular, a variation of the CAGE protocol using a single molecule sequencer [22] allows us to quantify transcription start site (TSS) activities at single base pair resolution from as little as approximately 100 ng of total RNA. We used this technology to capture transcription regulation across diverse biological states of mammalian cells in the Functional Annotation of Mammalian Genomes 5 (FANTOM5) project [23]. The collection consists of more than 1,000 human and mouse samples, most of which are derived from primary cells. This is a unique data set to understand regulated transcription in mammalian cell types. The broad coverage of biological states allows researchers to find samples of interest and inspect active genes or transcription factors in their biological contexts. The comprehensive profiling across the sample collection provides the opportunity to look up any gene, transcription factor or non-coding RNA of interest and to examine in which context they are activated across mammalian cellular states. CAGE-based TSS profiles at single base resolution allow the correlation of transcription activity with sequence motifs or epigenetic features. In previous studies, we generated TSS profiles based on CAGE in FANTOM3 [24,25] and FANTOM4 [26,27], but the diversity of biological states and the quantification capabilities were quite limited due to the state of the technologies at that point. To facilitate FANTOM5 data exploration from various perspectives, we prepared a set of computational resources, including a curated data archive and several database systems, so that researchers can easily explore, examine, and extract data. Here, we introduce the online resources with underlying data structure and describe their potential use in multiple research fields. This work is part of the FANTOM5 project. Data downloads, genomic tools and co-published manuscripts are summarized at [28].

## Results and discussions

### Annotation of the sample collection

In FANTOM5 [23], more than 1,000 human and mouse samples were profiled by CAGE. These include primary cells, cell lines, and tissues consisting of multiple cell types. To facilitate examination of the diverse and large number of samples by both wet-bench and computational biologists, we describe the samples from two complementary perspectives: (i) manual collection and curation of sample attributes and (ii) systematic classification using existing ontologies. Manual curation was accomplished via a standardized sample and file naming procedure based on a compiled set of sample attributes (such as age, sex, tissue, and cell type; details in Additional files 1, 2, and 3). Names are formed by concatenating the curated sample names (for example, 'Smooth Muscle Cells - Aortic, donor0'), RNA ID (for example, '11210-116A4') and CAGE library ID (for example, 'CNhs10838'), where the latter two enable us to track the samples in the form of RNA extracts and loaded sequencing materials (Additional file 4). Replicates are further identified with suffix notation (such as tech_rep#, biol_rep#, donor#, pool#) to the sample names. The resulting sample and file names are structured so that related samples (like developmental stages) will be grouped together in order when sorted alphabetically. We faced the challenge that the file names needed to be both informative for researchers and valid for computational systems that impose restrictions on the set of allowed characters in file names and file access paths. A full description of samples often requires a variety of symbols (for example, single quote in 'Hodgkin's lymphoma', slash, caret, parentheses in 'cell line:143B/TK^(−)neo^(R)'), and some computer systems have problems handling file names including these symbols. One option is to use short labels as in the case of genes, where unique short labels for human genes (called gene symbols) are determined through community discussions under coordination by the Human Genome Nomenclature Committee [29]. But we chose not to do this, as this introduces an extra layer of complexity in data handling and coordination, and an additional cognitive burden on human users. Instead, we decided to encode the sample names in 'URL encode' scheme (RFC3986) for file names, so that we can systematically generate them and decrease the risk of data tracing errors. This has the added advantage that URL path accessors to the files are consistent with those of the file system.

To classify samples systematically, we assembled the FANTOM Five (FF) Sample Ontology [23] consisting of the existing basic ontologies: cell types (CL), anatomical systems (UBERON), and diseases (DOID) [30-32]. We used the RNA ID as a unique identifier term (see Additional file 4 and below) of the individual samples and to link the corresponding FF ontology terms in a parent-child relationship. This scheme provides a way for researchers to query a group of samples based on existing knowledge and to aggregate related information systematically. In addition, we mapped graphical images in the BodyParts3D resource [33] to the UBERON terms composing the FF ontology, via the Foundational Model of Anatomy ontology [34]. This enables us to provide graphical shapes of individual organs in our databases.

### Overview of the data collected from the FANTOM5 samples

**Figure 1:**
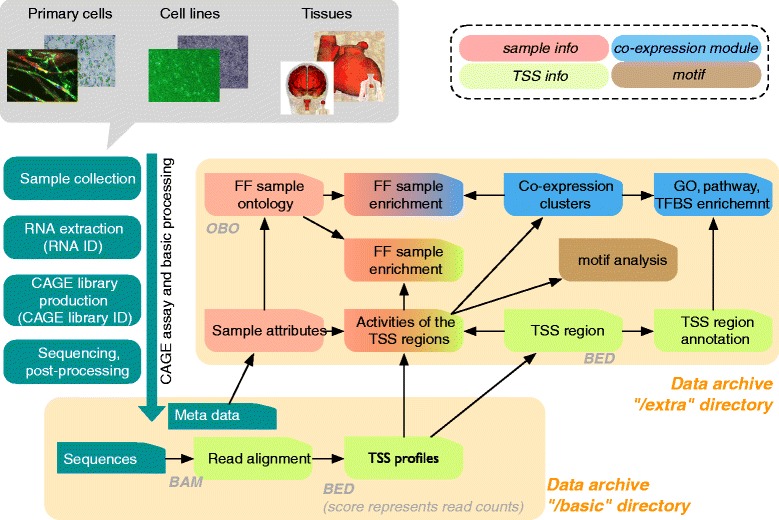
**FANTOM5 assay flow and the data archive.** Sample collection and data processing are indicated in a schematic view (green boxes). The resulting data and the analysis are collected into corresponding directories in the data archive (orange). GO, gene ontology.

The FANTOM5 analysis pipeline is shown in Figure 1, and resulting data types are summarized in Table 1. Cell or tissue RNA extracts were collected either from the FANTOM5 collaborators directly or purchased from companies. Each sample was assigned a unique RNA ID, annotated as described above, and CAGE libraries were constructed using either an automated system [35] or, for lower quantity RNA samples, a manual protocol [22]. Libraries were sequenced and analyzed (see Materials and methods) to generate TSS profiles for each sample and CAGE peaks were annotated with normalized expression level tags per million where library sizes were adjusted by relative log expression [36,37]. Further analyses resulted in quality assessment and promoter annotation, including gene association, gene ontology function, co-expression analysis and motif analysis. We also associate individual CAGE peaks with biological states where they are actively transcribed (see below), which was enabled by the systematic classification provided within FF Sample Ontology. We compiled these results as a consistent data set in a central data archive. The results of the standard processing pipeline are kept in a directory named ‘basic’, where all of the materials, data, and protocols are described in MAGE/ISA-tab [38,39]. The subsequent analysis results, such as the identified TSS regions, their quantified expressions, co-expression clustering, ontology enrichment and DNA motif analysis, are kept in a directory named ‘extra’.

**Table 1:** **Data files available in the data archive**

**Data or analysis type**	**Data format**	**Path**
Sample, RNA, and CAGE library information (metadata)	SDRF	/basic/*sdrf.txt
		/basic/*CAGE/00_*assay_sdrf.txt
Ribosomal RNA hitting reads	FASTA	/basic/*CAGE/*nobarcode.rdna.fa.gz (1,385 files)
Mapping results (including unmapped reads)	BAM	/basic/*CAGE/*nobarcode.bam (1,385 files)
TSS profiles (counts of obtained 5'-end reads at 1 bp resolution)	BED	/basic/*CAGE/*ctss.bed.gz (1,385 files)
Sample classification based on the FANTOM Five Sample Ontology	OBO	/extra/Ontology/ff-phase1-*.obo
CAGE peaks (TSS clusters)	BED	/extra/CAGE_peaks/*.bed.gz
CAGE peak annotation (descriptions and gene association)	OSC	/extra/CAGE_peaks/*.ann.txt.gz
Expression of the CAGE peaks	OSC	/extra/CAGE_peaks/*.osc.txt.gz
Co-expression clustering	OSC	/extra/Co-expression_clusters/*_co-expression_modules.tar.gz
*De novo* motif analysis	TXT	/extra/Motifs/novel_pwms.txt
Sample enrichment analysis	TXT	/extra/Sample_ontology_enrichment_of_CAGE_peaks /*.txt.gz
Gene ontology enrichment analysis of co-expression clusters	OSC	/extra/Co-expression_clusters/*co-expression_GOstats.tar.gz

### Interfaces to the series of FANTOM5 results

**Figure 2:**
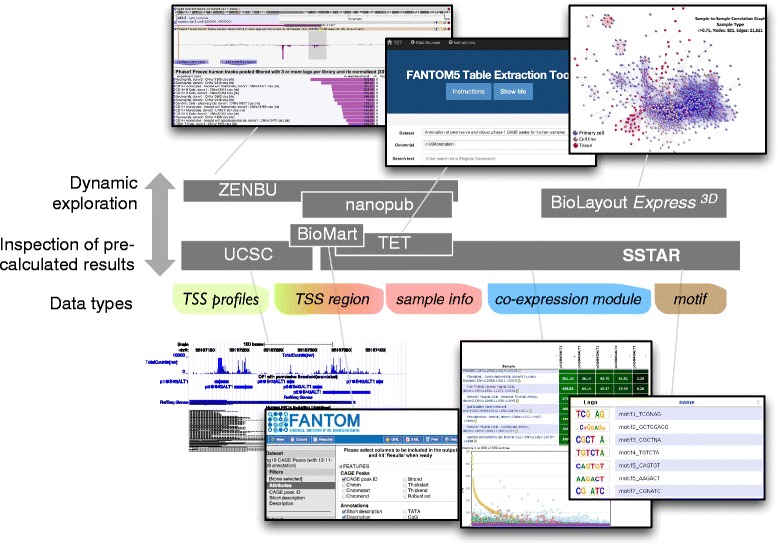
**Interfaces to FANTOM analyses.** Scope and contents of the database systems.

To provide these diverse data sets in a useful format for multiple purposes we created a series of database systems (Figure 2) that are complementary to each other in terms of hosted data or context. Researchers may be primarily interested in accessing data in two ways: (i) in-depth inspection of the computational characterization (analysis results) delineating cellular states, transcription initiation events and their regulation; and (ii) dynamic exploration of individual profiles (original data) on-demand. For in-depth inspection we made the comprehensive information accessible using existing and widely utilized software interfaces. For example, FANTOM5 tracks on the UCSC Genome Browser via track hub [40] allow users to inspect the FANTOM5 TSS regions together with epigenetic marks profiled by the ENCODE project [41] and Roadmap Epigenomics [42]. Our BioMart [43] instance makes it possible to export the annotation of CAGE peaks with a simple and stepwise interface. The Table Extraction Tool (TET) provides a simple way to obtain a relevant subset of expression intensities for individual CAGE peaks. The resulting expression tables downloaded from TET are formatted in a general expression matrix where rows represent CAGE peaks and columns individual samples, enabling users to immediately start their expression analysis without re-formatting. Additionally we created a semantic catalog of samples, transcription initiation and regulators (SSTAR); Abugessaisa *et al*., in preparation, a database system using the Semantic MediaWiki framework [44] to host the heterogeneous analysis results in a transparent way. Using SSTAR, researchers can access the FANTOM5 analysis results in a similar manner to Wikipedia [45] with a customized visualization and data export. From BioGPS [19], a gene annotation portal to study gene function, SSTAR entries for genes can be shown via its FANTOM5 SSTAR plugin. Further, we modeled the annotations and activities of CAGE peaks in the Resource Description Framework (RDF), published in a nanopublication format [46,47], and provided a set of SPARQL endpoints so that each of the peaks can be queried and cited by using Semantic Web technologies. A portion of the data stored in SSTAR is also loaded in RIKENBASE [48] to be associated with other RIKEN databases.

For interactive and dynamic data exploration, optimized for individual data types, we configured the ZENBU genome browser and analysis system [49], which stores and displays all CAGE experiments, including the genome alignments of individual CAGE reads as well as the annotation of each sample. It enables users to explore TSS activities in any region of the genome, with a user-selectable alignment threshold between the CAGE reads and the genome. The Enhancer Selector tool (Li *et al*., under preparation) stores the summarized activity profiles of the enhancers identified by CAGE [50] based on curated tissue categories and enables users to select a group of enhancers activated in specified conditions through its intuitive 'slider' interface. BioLayout *Express*^3D^ [51] presents the results of co-expression clustering as a three-dimensional visualization of expression space with an interactive user interface.

### Data exploration: use cases

All of the individual interfaces have their own scope and advantages and are linked to each other to allow easy access to relevant information. An example analysis flow using multiple tools is shown in Additional file 5, while a variety of explorations are possible for biological questions and hypotheses. Below, we provide examples to access FANTOM5 data via the specific interfaces.

#### Starting with sample details

**Figure 3:**
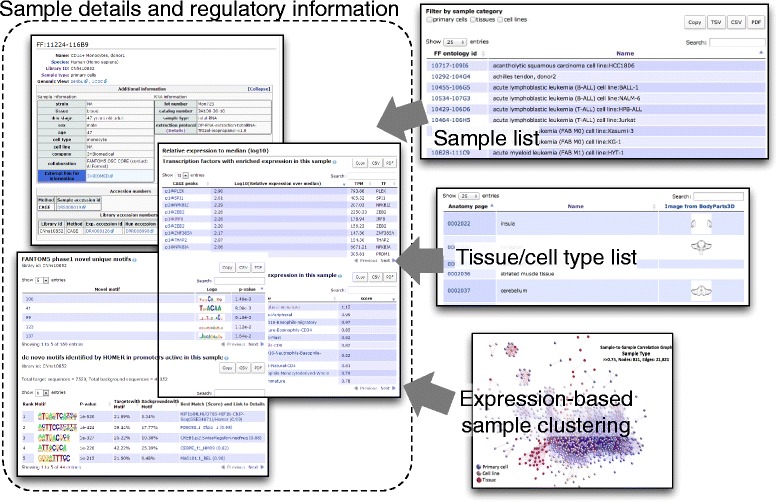
**Access to the sample details.** Detailed information of a sample, including regulatory information produced by computational analysis of its transcriptome, is summarized in a page (dotted box on the left). This page can be found by examining pages of listed samples or tissue cell types, or by looking at (dis)similarities of samples in a transcriptome space defined by expression clustering (right boxes).

Data exploration often starts from searching for samples of interest and examining details of the individual cellular states. SSTAR provides a collection of pages representing the complete sets of FANTOM5 samples, CAGE peaks, transcription factors and ontologies. It also contains analysis results such as expression and co-expression of peaks, enrichment scores, and motifs. SSTAR provides lists of samples profiled in FANTOM5 as individual sample pages (Figure 3) that store basic details such as donor age, sex, and RNA quality metrics as well as analysis results about transcription regulation, including relative expression levels of transcription factors and DNA binding motifs relevant in the sample. For example, a page corresponding to 'CD14+ Monocytes, donor 1' [52] shows essential transcription factors for monocytes sorted by relative abundance. *SPI1*, encoding the hematopoietic master transcription factor PU.1, for example, is ranked second (p1@SPI1). Its DNA binding motif, listed in the motifs section, is discovered by *de novo* motif analysis (PB0058.1_Sfpi1_1, Additional file 6).

#### Checking a group of samples based on manually curated classifications

SSTAR provides lists of the sample ontology terms (cell type, tissue, and disease ontologies) with hyperlinks to individual ontology term pages. Within each of these pages, detailed information on the term itself, such as cross-references and name spaces, are shown, and samples associated with the term based on FF Sample Ontology classification are listed (Figure 3; Additional file 7). The ontology term page also shows parent-children relationships via a graphical and interactive user interface by using the NCBO widget [53]. For example, a page describing the cell type 'monocyte' shows that it develops from promonocyte and into macrophage (Additional file 7). Furthermore, it shows the CAGE peaks highly active in the monocyte-related samples based on FF Sample Ontology Enrichment Analysis (Additional file 8).

#### Overviewing sample proximity and distance across transcriptome space

BioLayout *Express*^3D^ [51] is a powerful network analysis tool that provides an interactive way to explore similarity relationships between samples and transcription initiation activities (that is, CAGE peak expressions). The user can inspect a network in which nodes represent either samples or CAGE peaks where node colors are based on the co-expression cluster they belong to, and edges represent correlations between them above the user-defined threshold. The network displayed in a three-dimensional environment can be rotated, zoomed and explored interactively. Graphical representation of the FANTOM5 data allows the user to examine promoter expression patterns across nearly 1,000 samples included in this study or subsets thereof. A number of pre-calculated graph views (layout files) are available at our web resource. For example, a network shown in Additional file 9 enables us to examine sample-sample (dis)similarities, and one in Additional file 10 to examine relationships between CAGE peaks where their expression patterns can be displayed in a pop-up window. A web search function for nodes (samples or CAGE peaks) is set up to query the SSTAR or ZENBU databases for matches. For further in-depth examination, users can activate the clustering option based on the Markov Cluster Algorithm (MCL) [54] and adjust the parameters in order to obtain co-expression classes, or clusters, of samples sharing similar patterns in expression.

#### *Inspecting genes*, *transcription factors and DNA motifs*

**Figure 4:**
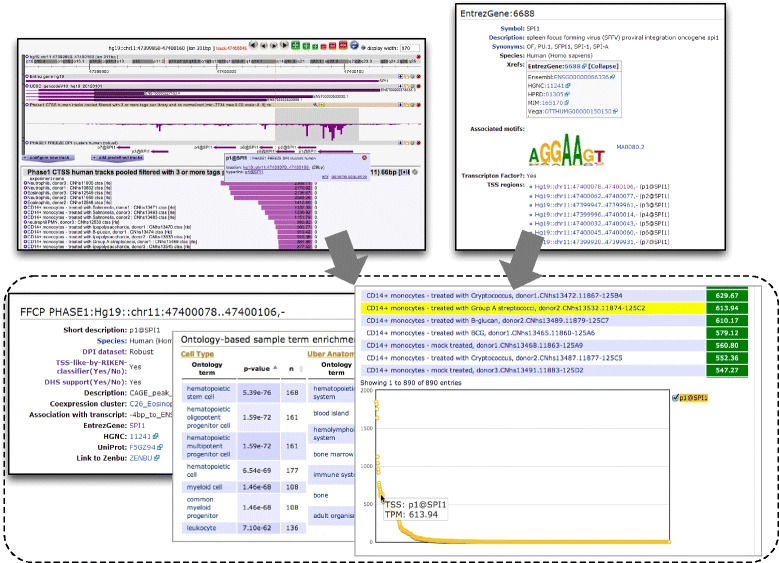
**Inspection of a transcription factor and its activity.** Detailed information on the most active CAGE peak at the *SPI1* promoter is summarized in one page (dotted box, bottom left). This page can be found by examining the *SPI1* gene on ZENBU (top left panel) or SSTAR (top right panel).

A simple keyword search of a gene in SSTAR (Additional file 11) allows us to find a gene page showing its associated CAGE peaks and its activity levels across all the samples, as well as basic gene information from EntrezGene [4]. For example, *SPI1* is associated with seven TSS regions whose expression profiles are summarized in a page as in Additional file 12. The hyperlinked 'TSS region' page shows further details, such as FF sample ontology enrichment analysis and the co-expression cluster it belongs to, as well as its activity profiles across samples (Figure 4). For genes encoding a known transcription factor, the gene page also includes its corresponding consensus recognition sequence ('DNA motif') if known. It shows the samples where transcription is significantly correlated with the motif occurrence (see Materials and methods) as well as its nucleotide pattern by sequence logo (Additional file 12).

#### Putting data in the genomic axis

ZENBU [49] provides an interactive interface to explore transcription initiation activities in their genomic context and it helps to examine transcription activity in-depth, independent of the CAGE peaks defined in FANTOM5 [23]. It also allows for selection of CAGE profiles to be displayed using the Data Explorer search tab (Additional file 13). A single ‘pooled’ track aggregating multiple CAGE samples allows a user to examine the expression profile in each of the CAGE profiles immediately by selection of any genomic regions. For example, selection of the *SPI1* promoter region in a pre-configured pooled track of all the FANTOM5 CAGE profiles displays accumulated transcription activities. From there one can apply a filter on sample names and sort by expression levels (Additional file 14). Several configurations prepared for the FANTOM5 data set are accessible from the ZENBU resource page. Similarly, we prepared a set of configured data files for the data hub in the UCSC Genome Browser [40], which allow users to overlay the FANTOM5 CAGE peaks and TSS profiles with the views and annotations maintained by the database management team and the community. For example, one can examine the CAGE peaks associated with *SPI1* and compare them with the ENCODE regulation tracks and segmentation tracks (Additional file 15).

#### Exporting selected data

Besides individual inspection of compiled results, further computational analyses with custom parameters and/or tools are sometimes required to build a working hypothesis and select candidates for experiments. Researchers can use several interfaces to obtain desired data rather than downloading and parsing large data files from the entire data archive. ZENBU and the UCSC Genome Browser both have export functions as a part of their user interface. In particular, ZENBU’s unique interface enables us to export expression profiles of arbitrary regions, which is useful for in-depth examination of non-annotated genomic regions. Similarly, portions of the data can be extracted using the BioMart [43] instance and TET tool. The former provides a way to select and obtain CAGE peak annotations, such as associated genes and promoter features, via a widely used interface (Additional file 16). TET lets users obtain a subset of data by specifying the desired columns and rows. In the FANTOM5 context, TET enables users to specify CAGE peaks and samples to be included. The resulting data matrix is immediately usable for expression analysis across CAGE peaks and biological samples (Additional file 17).

#### Connecting to linked data

In addition to data export in tab-delimited files, we also modeled the FANTOM5 data as nanopublications (the smallest unit of publishable information) [46,55]. Nanopublications expose individual records allowing automatic integration with any other linked data [56,57] and for citation tracking of their impact [58]. Each of the nanopublications is composed of three elements based on RDF (Additional file 18): an assertion (data or scientific statement), provenance for the assertion (how the assertion came to be), and publication information (how the nanopublication came to be). We have exposed three types of nanopublications from FANTOM5 data: CAGE peaks (type I nanopublications; see Materials and methods); their associated genes (type II); and their expression information (type III). By applying standard SPARQL [59] queries to the FANTOM5 nanopublications (available at [47]), specific results can be retrieved semantically. For example, Additional file 19 shows a SPARQL query to retrieve the samples related to skeletal muscle and activities of the TSSs for *MYOD*, a master regulator of myogenesis, in those samples. Although this is a simple biological question, automatic retrieval of its result is challenging due to ambiguities in several layers. For example, there are ambiguities in concept identification (*MYOD1*, not *MYOD*, is the official symbol in HUGO nomenclature), multiple CAGE peaks can be associated with the gene (actually four CAGE peaks are associated with *MYOD1*), and many different FANTOM5 samples, including cell lines and primary cells, are related to skeletal muscle but not all samples contain the keyword 'muscle' in the sample description (for example, myoblast). Despite these semantic complications, the query in Additional file 19 retrieves expected data (Additional file 20) by resolving these ambiguities with semantic integration of Linked Life Data [60], retrieved 16 April 2014) and the FANTOM5 nanopublications. We foresee that the nanopublications and associated SPARQL endpoints facilitate the automated integration with many other biomedical datasets.

### Continual evolution of resources to treat diverse sets of data

Based on our experience preparing the series of interfaces, here we discuss the challenges we faced in their preparation and the approaches we took, as a lesson for other future projects. At the initial stage of FANTOM5, we had a clear vision of the data set to be generated and analyses to be tackled, but we did not have a complete picture of the results, research questions and directions. The types of raw and processed data were clear, but it was difficult to determine the number of data files and data types, and to predict their complexities through the entire project.

Given the challenge of working with large amounts of data under such uncertainty, we started to prepare interfaces from a minimum set of visible tools requiring less data modeling assumptions ('data agnostic' tools). MediaWiki is designed for Wikipedia, a web-based, collaborative and flexible form of encyclopedia to collect a comprehensive summary from any branch of knowledge. Individual pages can contain any sort of description, and immediate data visibility on a page provides a means for data providers and generators to visually check, confirm or correct details, where Semantic MediaWiki extension helped us to retrieve relevant information even if stored in different pages. Genome browsers require data to have genomic coordinates, and the use of genome browsers for inspection of data (in the context of other data in the same genomic region) is obviously important for the genomics field. Loading all the CAGE profiles into ZENBU helped us to validate the processing of samples by checking the expression of marker genes. After starting with these two interfaces, we gradually added other interfaces to complement uncovered parts. We included BioMart, BioGPS plugin, and UCSC DataHub to disseminate our results across these user communities, and introduced the enhancer selector, BioLayout and TET to facilitate further analysis and inspection of our resources. This might serve as a practical approach in treating data for exploratory research, and a guide for developers to design tools and their functions.

## Conclusions

In FANTOM5, the FANTOM Consortium has profiled TSS level transcription activities in a diverse range of samples. We assembled the data and analysis results into an on-line resource containing a comprehensive expression atlas for exploration from multiple perspectives. The expression atlas covers the largest number of samples (nearly 1,000 human and 400 mouse samples) based on HeliScopeCAGE [22]. An existing expression resource, BioGPS [19], and one of the most popular databases for microarray-based gene expression atlases, provides around 200 samples at its most recent version. CellMontage, a system for searching gene expression databases based on profile similarity, exhaustively collected hundreds of thousands of human microarray gene expression profiles from different public repositories, providing a tool to retrieve data sets from different studies and laboratories [61]. Our resource uniquely consists of the largest number of samples on a single platform. In terms of TSS profiles, the FANTOM5 collection is the largest (ENCODE profiled 36 cell lines by CAGE [41], while the DataBase of Transcriptional Start Sites (DBTSS) [62] has TSS profiles from 20 tissues and 7 cell lines). The FANTOM5 atlas expands the existing resources in terms of coverage and diversity of samples that were profiled. Moreover, considering the nature of HeliScopeCAGE data, absolute measurement of capped RNA abundance by using a single molecule sequencer can achieve higher quantification ability [63] compared with the previous CAGE technology employing two steps of PCR [64]. Thus, the FANTOM5 atlas could contribute to the research community by providing high quality data.

The resource provides extensive annotation about transcription initiation as well as cellular transcription states, which is far beyond merely assembling profiles. We strategically defined TSS regions in a data-driven manner and annotated them by performing a series of computational analyses. Such analyses enriched the characterization of experimentally defined regions, although they also increased data types. We prepared a series of database systems to host heterogeneous data to make it possible for researchers to explore the data from multiple perspectives. The tools or database systems shown in Figure 2 provide multiple means to play with data interactively, export only a subset of the entire data, and integrate with other data beyond FANTOM5. In the on-going activities of the second phase of FANTOM5, we are now working on time-dependent dynamics and their regulation. We expect additional data types and are going to expand the collection to cover additional analysis.

## Materials and methods

### A standardized description of samples and experimental conditions

A wide range of RNA samples with different origin and with replicates was produced for FANTOM5. To describe, in a consistent manner, the entire set of samples, experiments, and protocols, we employed the MAGE/ISA-tab file format [38,39], a standard format to describe experimental details. The experimental steps described in the file can be visualized with SDRF2GRAPH [65], a tool developed during the FANTOM4 project [26] (available as a web tool at [66]), providing an intuitive representation of the complex experimental steps. These meta-data files help to document the data structure of the FANTOM5 project and support its use and biological interpretation.

### Standardized data collection, quality control and automated data processing

For each FANTOM5 sample, cDNAs resulting from CAGE library preparation were loaded onto HeliScope flow cells. Each sequencing result was then systematically processed, discarding sequences that are too short or that represent artifacts [67], aligning the obtained reads to the reference genome sequences [68], and counting CAGE read alignments based on their 5’ end (termed CAGE tag start site (CTSS) [25]) with required mapping quality ≥20 and sequence identity ≥85%. Mapping files were first filtered to discard bad alignments and then indexed by using SAMtools utilities [69] to allow both extraction of specific mapping locations and access the BAM files remotely. The mapping files were then converted into CTSS BED files using a combination of BedTools [70] and shell commands to reduce the data. They were then systematically named using a combination of sample names and unique identifiers (Additional file 4). This yields a quantification of transcription initiation activity in each sample at single base pair resolution.

Based on the TSS profiling data above, we determined TSS regions by calling peaks over the CAGE signals (Additional file 21) [23]. We refer to them as 'CAGE peaks' to avoid confusion with co-expression clustering below. We assigned peak names based on the closest gene (located within 500 bp upstream of the 5’ end of the gene model, or alternatively on its first exon up to 500 bp downstream), and ranked them based on the CTSS counts when multiple CAGE peaks were associated with the same gene. For example, p1@B4GALT1 (CAGE peak 1 at the B4GALT1 5’ end) indicates a peak near the B4GALT1 gene which is the most highly expressed among those associated with the same gene. Further, we examined the association of CAGE peaks with gene structure and repetitive elements based on a curation rule (see below). We also examined the similarity of their neighboring genomic sequences to conventional TSSs by a machine learning approach to distinguish TSS-like sequences from others [23]. We quantified activities of the identified TSS regions based on the counts of CAGE read alignments as tags per million after adjusting the library size by the relative log expression method [36,37].

Based on the TSS regions and their expression levels, we performed co-expression analysis by applying the MCL [23,71] followed by pathway enrichment analysis (Figure 1). Gene ontology enrichment analysis [72] allowed us to annotate individual co-expression clusters in terms of gene function, while the sample ontology let us annotate the biological context in which a CAGE peak or a co-expression cluster is activated in an analogous way to gene set enrichment analysis [73]. In parallel, we examined the presence of DNA motifs, which are regulatory elements encoded in the genome. We examined over-representation of known DNA motifs (obtained from Jaspar [74]) in each of the co-expression clusters, and correlation between their presence and expression (see Materials and methods). Furthermore, we explored novel DNA motifs by evaluating their correlation with CAGE expression patterns [23].

### Significance assessment of DNA motifs

We predicted putative transcription factor binding sites (TFBSs) using a position-weight matrix model as implemented in Biopython [75] for each JASPAR [74] motif and for each novel motif, with a background probability based on a 40.9% GC content. The position-weight matrix scores were converted to Bayesian posterior probabilities using a prior probability of 5 × 10^-4^. We retained all predicted TFBSs with a posterior probability larger than 0.1. We then associated predicted TFBSs with the 184,476 (human) or 116,064 (mouse) robust promoters [23] as described previously [26] using a -300.. +100 bp region with respect to the representative genome position of the promoter, defined as its most highly expressed position in the FANTOM5 samples. For each motif in each sample, we calculated the Pearson correlation across the robust promoters between the number of TFBSs estimated for each promoter and its CAGE expression level. For each motif, we repeated this procedure for 1,000 randomized position-weight matrices, in which the order of rows (corresponding to positions along the motif) is randomly permuted. We then expressed the Pearson correlation for each motif as a Z-score by subtracting the mean and dividing by the standard deviation of the Pearson correlations found for the randomized motifs. The *P*-value displayed is the tail probability of the normal distribution corresponding to this Z-score.

For each novel motif, we calculated the number of predicted TFBSs for each promoter by summing their posterior probabilities. We averaged this number over the robust promoters and multiplied it by the number of robust promoters in each of the co-expression clusters to find the expected number of TFBSs for the motif under the null hypothesis that the motif is not overrepresented in the given co-expression cluster. The observed number of TFBSs of a motif was found by summing its predicted TFBSs over the co-expression cluster. We then calculated the statistical significance of motif overrepresentation in the co-expression cluster by finding the tail probability of the observed number of TFBSs under a Poisson distribution with a mean equal to the expected number of TFBSs in the co-expression cluster.

### Annotation of CAGE peaks based on transcript structure

We devised a hierarchical approach to annotate TSS regions (or CAGE peaks) with respect to Gencode V10 transcript model structures such as TSSs, proximal promoter regions (500 bp upstream and 500 bp downstream of the TSS, or ending with the 3' end of its first exon), exonic region split into coding and non-coding (differentiating non-coding transcript exons, coding transcripts' 5' UTR and 3' UTR exonic regions) as well as relative position within the transcript (first, inner or last exon of the transcript), and intronic regions (similarly differentiated with respect to the coding sequence and position relative to the transcript). We also defined genome segments corresponding to the opposite DNA strand of those TSSs, proximal promoters, exons and intronic regions. A CAGE peak can overlap more than one genome segment region (for example, the proximal promoter region of a transcript and the first intron of another co-localized transcript). The annotation follows this hierarchy: TSS followed by proximal promoter regions, first followed by inner and last exons, antisense the TSS, then proximal promoter regions, then exonic regions, and finally intron (first sense and then antisense). The complete process is described in Additional file 22, and its implementation is based upon BedTools IntersectBed and groupBy utilities [70].

Finally, we used the same genome segmentation annotation pipeline to annotate CAGE peaks with respect to CpG island proximal region (retrieved from the UCSC table browser), TATA box proximal region (based on a genome-wide scanning of the JASPAR TATA-binding protein position weight matrix [74]), repeat elements (retrieved from the rmsk UCSC table) and ENCODE clustered TFBS proximal region (wgEncodeRegTfbsClustered track from UCSCwgEncodeRegTfbsClustered track from UCSC; region defined as cluster boundaries ±300 bp).

### ZENBU data load and view configuration

We implemented a semi-automated pipeline using command line tools for bulk loading of the large numbers of CTSS and BAM alignment files into ZENBU along with the corresponding sample annotation metadata using ZENBU's command line tools [49]. Several preconfigured views where created and updated to aid users in their research activities. Views included full sets of human and mouse samples, together with primary cell only, cell line only and tissues only. In addition, the flexibility of ZENBU allows researchers to modify and create their own visualization views on the FANTOM5 data and share them publicly or within a collaboration.

### BioMart interface for the defined transcription start site regions

BioMart [43] is a freely available, open source, and powerful query-oriented data management system. The BioMart system provides simple web browser interfaces and web services that allow a user to rapidly access an underlying database without knowledge of its data model. We customized the BioMart system to have CAGE peak annotation data and sample annotation data for both human and mouse. The FANTOM5 BioMart provides researchers with a simple web interface for performing queries of the FANTOM5 CAGE peaks and samples. It holds 1,048,124 human and 652,860 mouse CAGE peaks for 889 human and 389 mouse samples. Each CAGE peak has multiple attributes representing various annotation properties, including gene association, repeat association, robust and permissive designations, TSS-like flags, and GENCODE association for human and Ensembl association for mouse.

### Configuration of BioLayout

BioLayout *Express*^*3D*^ is an application that has been specifically designed for the integration, visualization, and analysis of large network graphs derived from biological data. It can be configured to a high degree in order to respond to the needs of various areas of research. The FANTOM5 BioLayout runs on a Java webstart program accessible from the FANTOM5 site. When the Java webstart application is launched BioLayout is opened with the input files that have been chosen as a default view describing our data collection. Nodes can be either samples or genes. BioLayout itself can be configured in order to provide access to other tools, such as SSTAR sample/gene searches or ZENBU experiment searches.

### Table extraction tool

FANTOM5 expression data are primarily distributed in compressed tab-separated-value (TSV) file format, each file consisting of the full set of CAGE peaks (184,827 rows in human and 116,277 rows in mouse) and expression values over samples (975 columns in human and 399 columns in mouse). In order to assist in the data extraction process we have created the FANTOM5 Table Extract Tool (TET). TET is intended to be a simplified way of extracting relevant sections from a curated set of FANTOM5 data tables. Using TET a user will select one of the FANTOM5 data sets, select the columns they wish to extract (that is, samples), then specify a set of rows (that is, CAGE peaks) using a regular expression search pattern, and finally view or download the resulting subset.

### Nanopublication

When exposing nanopublications from FANTOM5, we followed a four-step process as in Additional file 23. First, we examined the dataset to identify conceptual entities (for example, CAGE peaks, TSSs, genes) and assigned appropriate ontological descriptors. Second, we composed RDF triples and used the Vocabulary of Interlinked Datasets (VoID) [76] to create a ‘naive’ data model describing the data structure of the FANTOM5 entities. Using VoID statements, we could convert the dataset to 'nanopublication compliant' RDF and give each entry in the dataset (for example, each row-column combination) a Uniform Resource Identifier (URI). For example, each row of the dataset is transformed to a CAGE peak web resource. Using the void:inDataset predicate, each CAGE peak is linked back to the resource for the entire dataset. Subsequent predicates connect the CAGE peak to entities that represent columns of the raw dataset.

The third and most intellectually demanding step was to model the scientifically meaningful associations, the provenance metadata and publication information. This step uses the framework of the naive model to construct the actual nanopublication data model. When considering the FANTOM5 dataset, we developed several compelling proposals on how to model TSS-related assertions. As we worked through the models, we concluded that gene association should be a separate assertion (that is, a separate nanopublication) from the definition of a CAGE peak region as well as its expression. We generated three types of nanopublications: type I nanopublications make the link between CAGE peaks and the physical genome location; type II nanopublications make explicit the association that a particular CAGE peak is also a TSS region for a particular gene; type III nanopublications link the CAGE peaks to samples (that is, species, cell type) with the expression levels in those samples. This has several advantages: first, the process used to determine gene association is an independent process from the identification of CAGE peaks, so the provenance of gene association should be different from CAGE peak identification. Second, by separating the gene association from CAGE peak assertion, we can easily release a new set of associations if the FANTOM consortium needs to repeat the gene association process with different sets of data and/or parameters without redefining CAGE peaks. Third, it increases the granularity and reusability of data as others may use their own method/data to assign gene associations with FANTOM5 CAGE peaks. In modeling the provenance and publication information elements of the nanopublications, we chose here minimal models that simply referenced the FANTOM5 Consortium. As they are used in this study, the nanopublications have a clear provenance and so the minimal model is sufficient and without unnecessary complications. However, as stand-alone publications the provenance could be elaborated upon, creating more ‘autonomous’ data with distinct advantages for maximizing citations or for tracking scientific impact.

Lastly, we applied each of the three developed nanopublication models to instantiate the individual nanopublications as a referenceable linked data resource. This involved writing a script to instantiate the triples that compose the nanopublications. These triples were initially exported as large RDF files, which were then uploaded in the triple store provided by the Database Center for Life Science (DBCLS). The triple store is an OpenLink Virtuoso OS 7.1 and provides the SPARQL endpoint that is required to do integration queries such as the one shown in the section above. The last step consisted of making the nanopublication URLs resolvable, which is encouraged by and in line with the principles of Linked Data. This was achieved by means of a virtual host redirect on the Apache web server and a small application to query the triple store and return the requested nanopublication as serialized RDF (in Trig format. An example of each type of nanopublication, as well as a direct link to the triple store is available at [47]).

In writing these nanopublications, we surveyed existing ontologies. However, these were inadequate for our purposes and we decided to develop our own ontology, such as Reference Sequence Annotation (RSA) to fill the gap [77]. We wanted the RSA to accommodate the basic CAGE region description as well as scenarios such as allowing a single annotation to be mapped onto different reference assemblies. This provided the mechanism to compare data between FANTOM4, FANTOM5, and others.

### Computational resource

To provide the on-line resources for FANTOM5, we used nine physical servers and one virtual server for web applications, databases and file systems (not including the RDF store, Enhancer Selector tool and RIKENBASE). We used in total approximately 120 Tbytes hard disk space for storing data. We used existing software to host the data, and URLs of the source code are summarized in Additional file 24. All of the data are available at [28].

## Additional files

Additional file 1:
**Attributes collected for individual samples.**
Additional file 2:
**Curated names for human samples.**
Additional file 3:
**Curated names for mouse samples.**
Additional file 4:
**Structure of file names. (A)** File names are organized in a systematic way, where sample names, CAGE library ID, RNA ID, and other information are delimited with dot ('.'). To allow handling of special symbols by computers (such as Unix), the sample names are encoded by URLencoding. **(B)** An example code to decode the sample names in R. Additional file 5:
**An example of analysis flow.** Analysis steps are indicated in rounded boxes, supplemented with tool names used. On the right side, analysis examples at each step for someone who is interested in transcriptional regulation networks to implement monocytic function in fibroblasts (as in [78]) are shown. Additional file 6:
**Information and analysis results on a monocyte profile.** The information collected on the samples, like detailed sample and RNA information, highly expressed transcription factors, significant *de novo* motifs, co-expressed sample clusters, and highly expressed repeats are summarized into a single SSTAR page. Additional file 7:
**Access to individual samples in SSTAR.** Two ways to identify samples of interest in SSTAR: by using a list of sample names (orange arrows); by cell type inspection followed by selection of samples of interest (purple arrows). If the sample of interest has one UBERON term associated with it, a search through tissue types can be performed too (blue arrow). Additional file 8:
**Sample ontology enrichment analysis connected to CAGE peak expression.** Results of sample ontology enrichment analysis on 'hematopoietic cell' showed one of the *SPI1*-related CAGE peaks (p6@SPI1) as enriched. A link to the CAGE peak page where its individual expression pattern can be confirmed. Additional file 9:
**Graphical representation of sample-sample relationships in the transcriptome space by BioLayout** ***Express*** ^***3D***^ **.** Individual nodes (spheres) indicate a sample in the transcriptome space where the MCL (Markov cluster algorithm)-based clusters of samples are represented. Clustering is obtained by using correlation coefficients of expression as proximity metric. The three-dimensional graphs can be zoomed in/out and rotated by mouse operations such as dragging. Additional file 10:
**Graphical representation of CAGE peak relationships in the transcriptome space by BioLayout** ***Express*** ^***3D***^ **.** Individual nodes (spheres) indicate a CAGE peak or a group of CAGE peaks (cluster) very close to each other in the transcriptome space where MCL-based clusters of CAGE peaks are represented. Clustering is obtained by using correlation coefficients of expression as proximity metric. Expression patterns of each CAGE peak can be shown as a graph by pressing the Ctrl key followed by left-mouse button click. Additional file 11:
**Find genes by keyword search.** Keyword search in the SSTAR top page enables genes to be found. Additional file 12:
**Access to transcription factors and DNA motifs.** The side bar menu (top left) provides links to lists of transcription factors and DNA motifs. A gene page for a transcription factor (on the right) shows detailed information, including binding motifs. A DNA motif page (center) provides a list of associated samples (the center window). Additional file 13:
**ZENBU Data Explorer.** The upper panel shows the data explorer tab and the available options for displaying all data sets (preconfigured views, preconfigured tracks, experiments, annotations). The lower panel is an example of expression experiments where all data sets are listed, including FANTOM5 CAGE. Users can select multiple data sets for individual or pooled graphical representation. Additional file 14:
**Interactive inspection of TSS activities with ZENBU.** The upper panel displays graphical representation of CAGE signals at the *SPI1* locus along the genome. Mouse dragging operation enables a genomic region of interest to be specified (dark grey), and the expression intensities under the region are dynamically visualized (lower panel). The representation can be configured by clicking the 'gear' icon. Additional file 15:
**FANTOM5 TSS regions associated with ENCODE regulatory track on the UCSC Genome Browser.** FANTOM5 data hub allows the FANTOM5 data to be displayed on the UCSC genome browser. In addition to CAGE peaks displayed in this figure, CAGE signals along the genome for individual experiments can be selected. Additional file 16:
**Annotation export with BioMart.** This screenshot shows an example of how to obtain annotations of CAGE peaks, including short descriptions, Human Genome Nomenclature Committee gene IDs, presence of a TATA-box and CpG content. Additional file 17:
**Table Extraction Tool.** An example of how to export a subset of CAGE peak expression values using TET. Users can select columns in an interactive manner as shown in the left panel, and select rows by specifying the matching string (regular expression). The result can be exported as a table (the right panel) or visualized as a heat map. Additional file 18:
**Schema of the annotation pipeline.** A nanopublication is a schema built on top of existing semantic web approaches that essentially labels a single scientifically meaningful (publishable) assertion with metadata such that individual assertions are citable and their impact trackable. Nanopublications are composed of three elements: (1) the Assertion; (2) the Provenance metadata of the assertion (for example, authors, methods, funding source, date/time); and (3) the Provenance metadata about the nanopublication itself, in this case called Publication Info. Additional file 19:
**An example of a SPARQL query.** A SPARQL query that integrates data from three different Linked Data resources: the FANTOM5 nanopublication repository, the FANTOM5 Ontology and Linked Life Data. **(A)** The variables in the query linking the different datasets together. First the FANTOM5 ontology is queried to find samples from skeletal muscle. Then Linked Life Data is used to link the given gene symbol *MYOD* to a Bio2RDF resource URL. This Bio2RDF URL is used in the type II nanopublications to identify the CAGE peaks, which are a TSS region for the given gene. Using the type 3 nanopublications, we restrict the search for TSSs to the previously identified sample types that have a tags per million value larger than 0 (meaning that there is evidence for transcription on that region). Finally, the type I nanopublications provide the start and end coordinates for the TSSs. **(B)** The actual query. Additional file 20:
**An example of a SPARQL query.** Retrieved data for the query in Additional file 19 is shown. Additional file 21:
**CAGE peaks and their annotation.** Examples of CAGE peaks identified in FANTOM5. Six peaks in the proximal region of *B4GALT1* promoters are identified, and their names are indicated as p#@B4GALT1. The track below indicates that all of the peaks are supported by at least one EST (expressed sequence tag) model. Additional file 22:
**Classification of CAGE peaks according to the transcript structure.** Our hierarchical approach annotates CAGE peaks (left side, colored boxes) with respect to Gencode V10 transcript model structures (right side, grey boxes). The output of one step represents peaks that were not annotated yet and makes the input to the next step, as indicated by the direction of the arrows. The hierarchy is first run for sense transcript models, and then again for anti-sense ones. At the end of the pipeline, peaks are annotated as upstream and downstream (first sense, then antisense) of a TSS. Additional file 23:
**Workflow converting FANTOM5 data into nanopublications.**
Additional file 24:
**URLs of the source code used in the gateway.**

## Abbreviations

bp: base pair
CAGE: cap analysis of gene expression
CTSS: CAGE tag start site
FANTOM5: Functional Annotation of Mammalian Genomes 5
FF: FANTOM Five
GO: Gene Ontology
MCL: Markov Cluster Algorithm
PCR: polymerase chain reaction
RDF: Resource Description Framework
SSTAR: semantic catalog of samples, transcription initiation and regulators
TET: Table Extraction Tool
TFBS: transcription factor binding site
TSS: transcription start site
UTR: untranslated region
