# Supplementary material for: Gateways to the FANTOM5 promoter level mammalian expression atlas
Source: Genome Biol. 2015 Jan 5;16(1):22. doi: 10.1186/s13059-014-0560-6 (PMC4310165; doi:10.1186/s13059-014-0560-6)
Supplement: Additional file 4: — Structure of file names. (A) File names are organized in a systematic way, where sample names, CAGE library ID, RNA ID, and other information are delimited with dot ('.'). To allow handling of special symbols by computers (such as Unix), the sample names are encoded by URLencoding. (B) An example code to decode the sample names in R. [file 13059_2014_560_MOESM4_ESM.pdf]

(A)

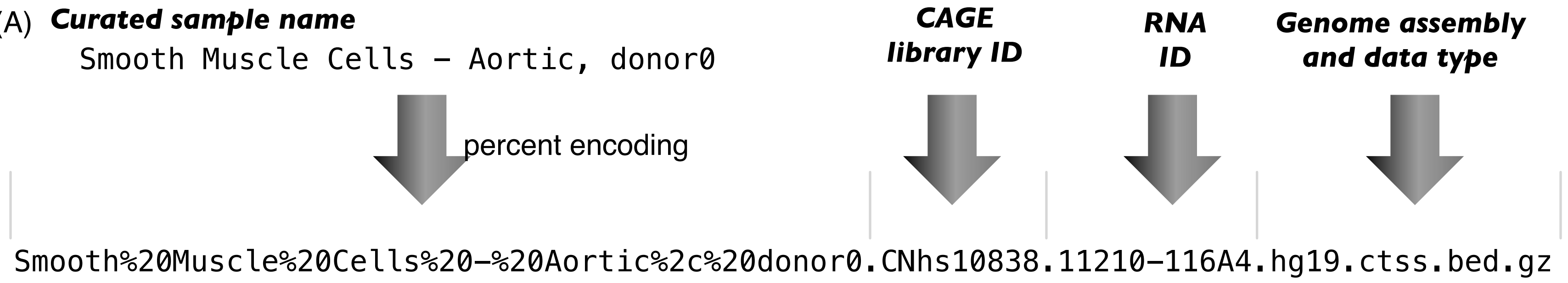

(B)

```
R> SAMPLE_NAME = "Smooth%20Muscle%20Cells%20-%20Aortic%2c%20donor0.CNhs10838.11210-116A4.hg19.ctss.bed.gz"
R> URLdecode( strsplit(SAMPLE_NAME,"\\.")[[1]][1] )
[1] "Smooth Muscle Cells - Aortic, donor0"
```
