# Supplementary figures and images for: Gateways to the FANTOM5 promoter level mammalian expression atlas
Source: Genome Biol. 2015 Jan 5;16(1):22. doi: 10.1186/s13059-014-0560-6 (PMC4310165; doi:10.1186/s13059-014-0560-6)

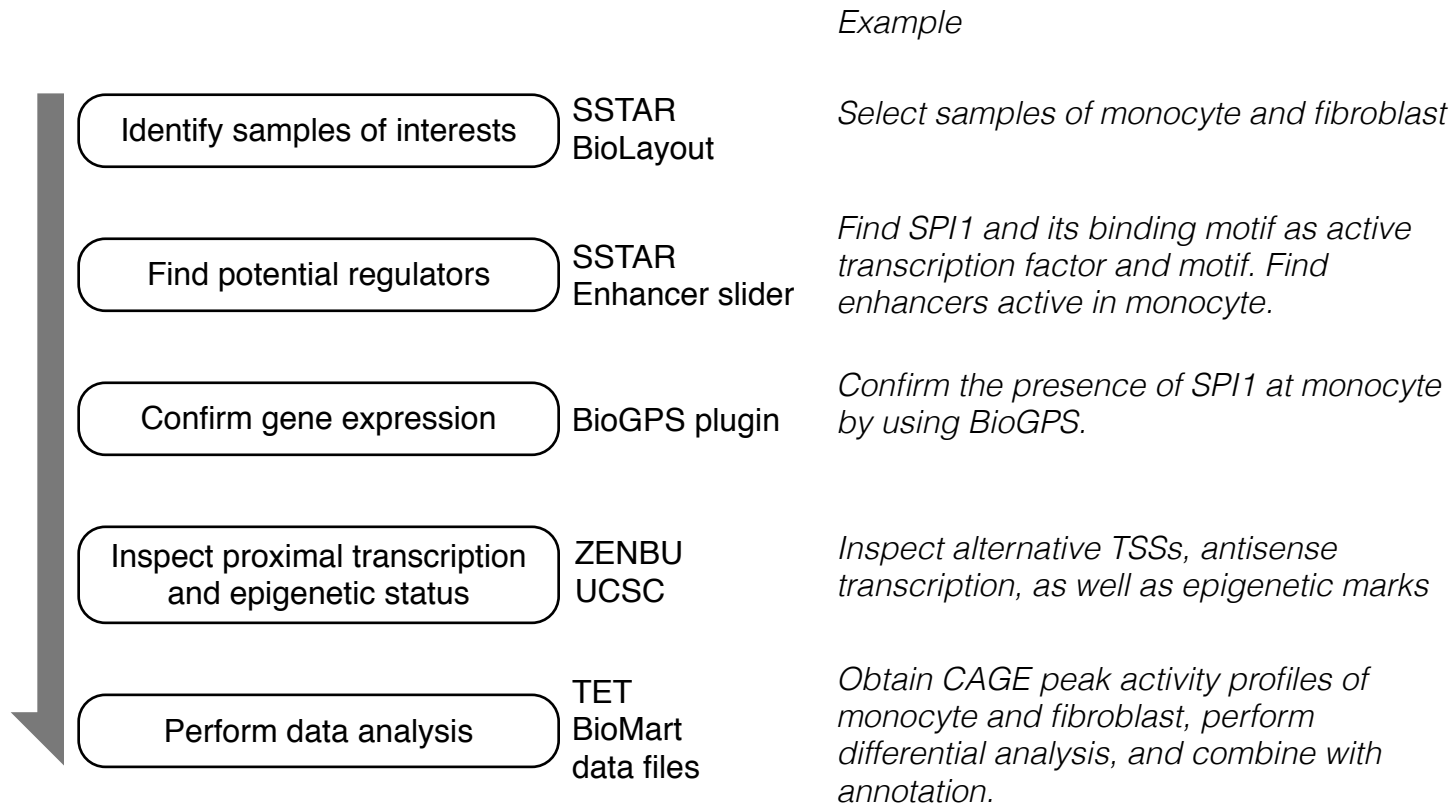

Supplement: Additional file 5: — An example of analysis flow. Analysis steps are indicated in rounded boxes, supplemented with tool names used. On the right side, analysis examples at each step for someone who is interested in transcriptional regulation networks to implement monocytic function in fibroblasts (as in [78]) are shown. [file 13059_2014_560_MOESM5_ESM.pdf]

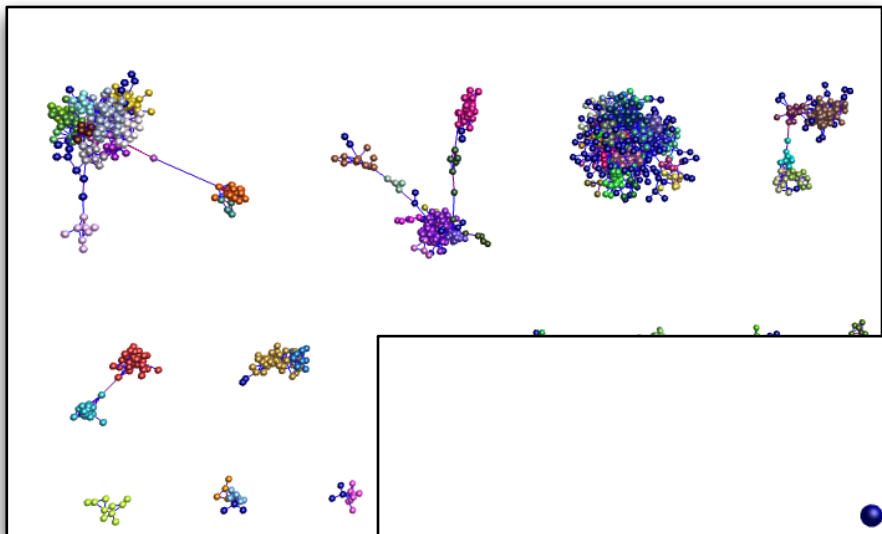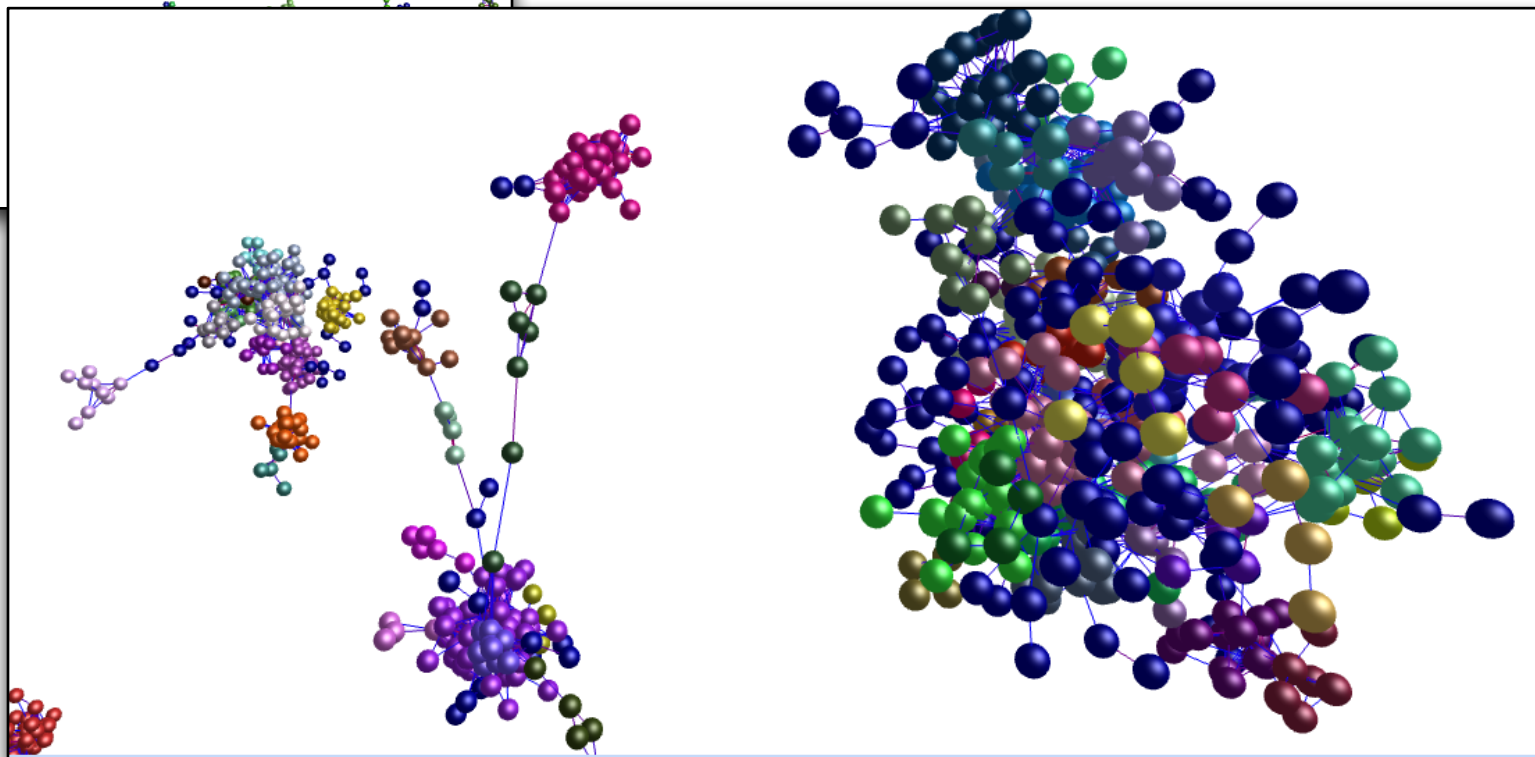

Supplement: Additional file 9: — Graphical representation of sample-sample relationships in the transcriptome space by BioLayout Express 3D . Individual nodes (spheres) indicate a sample in the transcriptome space where the MCL (Markov cluster algorithm)-based clusters of samples are represented. Clustering is obtained by using correlation coefficients of expression as proximity metric. The three-dimensional graphs can be zoomed in/out and rotated by mouse operations such as dragging. [file 13059_2014_560_MOESM9_ESM.pdf]

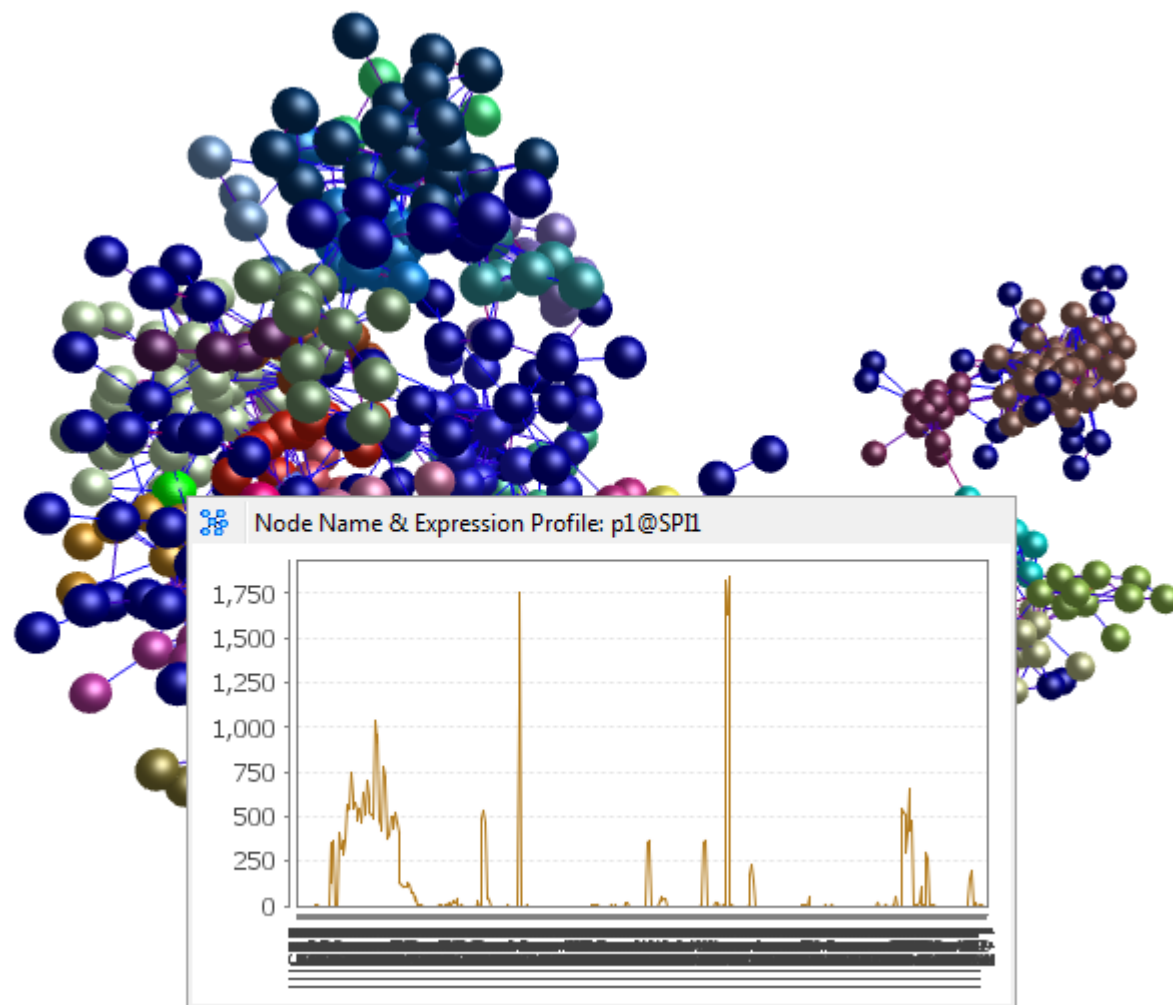

Supplement: Additional file 10: — Graphical representation of CAGE peak relationships in the transcriptome space by BioLayout Express 3D . Individual nodes (spheres) indicate a CAGE peak or a group of CAGE peaks (cluster) very close to each other in the transcriptome space where MCL-based clusters of CAGE peaks are represented. Clustering is obtained by using correlation coefficients of expression as proximity metric. Expression patterns of each CAGE peak can be shown as a graph by pressing the Ctrl key followed by left-mouse button click. [file 13059_2014_560_MOESM10_ESM.pdf]

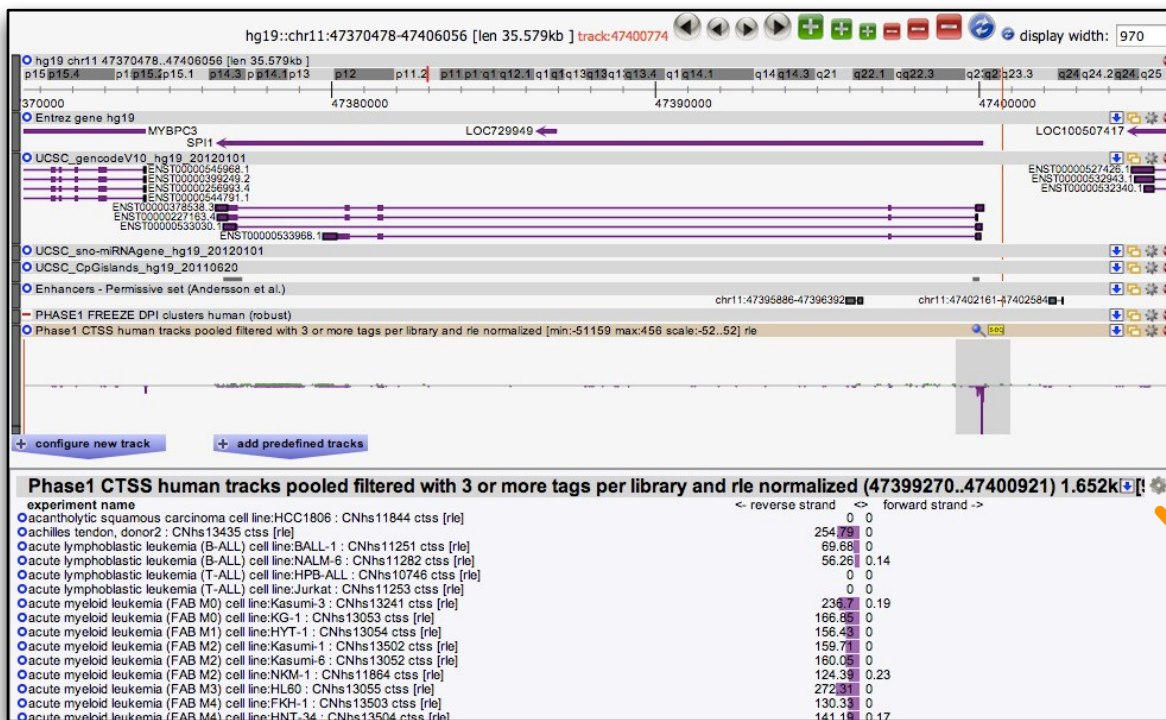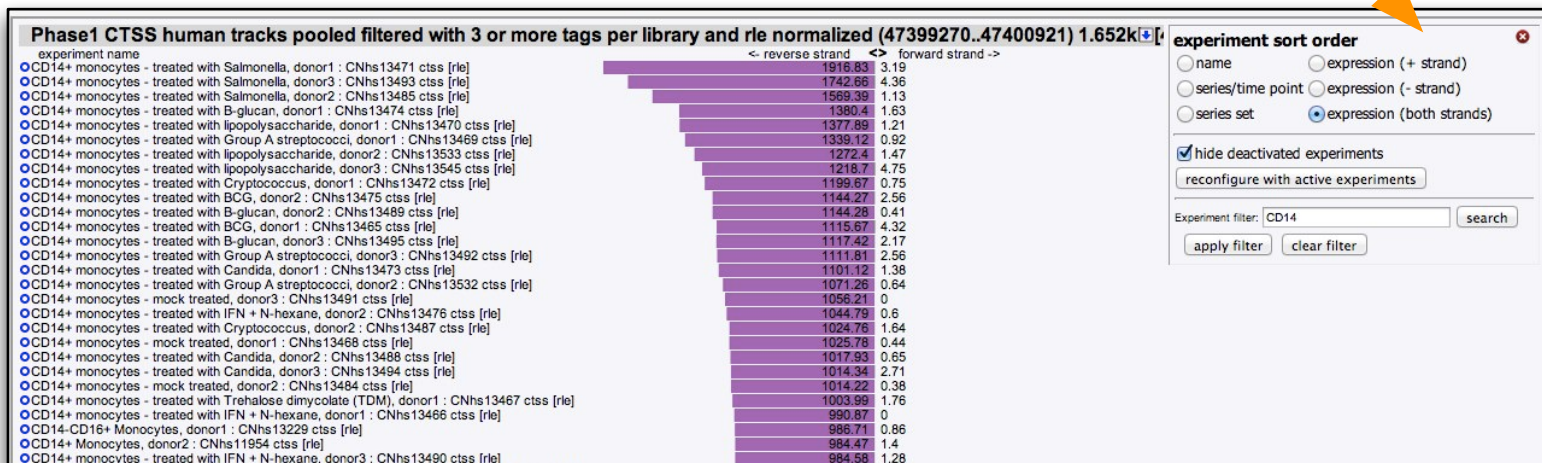

Supplement: Additional file 14: — Interactive inspection of TSS activities with ZENBU. The upper panel displays graphical representation of CAGE signals at the SPI1 locus along the genome. Mouse dragging operation enables a genomic region of interest to be specified (dark grey), and the expression intensities under the region are dynamically visualized (lower panel). The representation can be configured by clicking the 'gear' icon. [file 13059_2014_560_MOESM14_ESM.pdf]

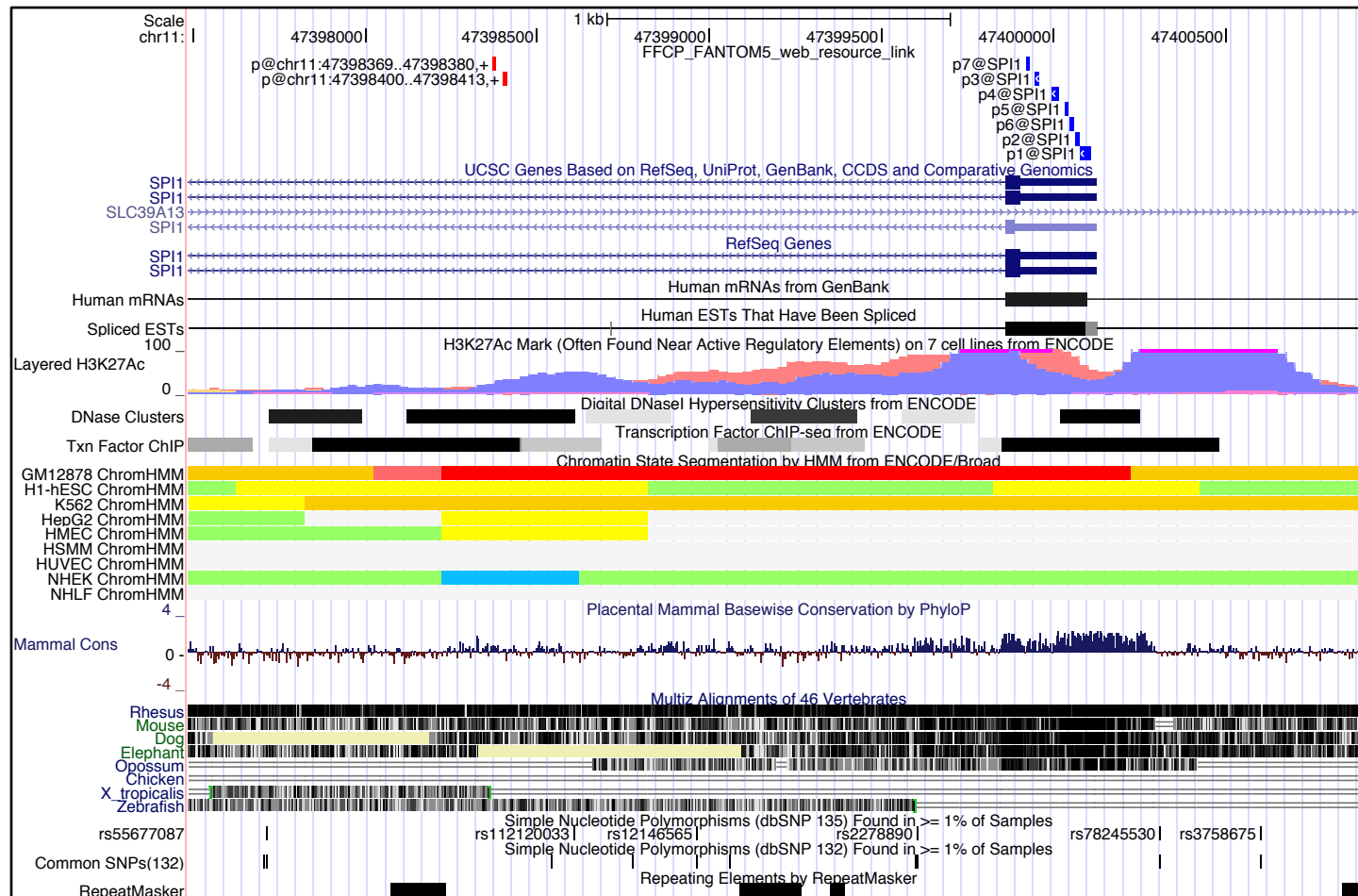

Supplement: Additional file 15: — FANTOM5 TSS regions associated with ENCODE regulatory track on the UCSC Genome Browser. FANTOM5 data hub allows the FANTOM5 data to be displayed on the UCSC genome browser. In addition to CAGE peaks displayed in this figure, CAGE signals along the genome for individual experiments can be selected. [file 13059_2014_560_MOESM15_ESM.pdf]

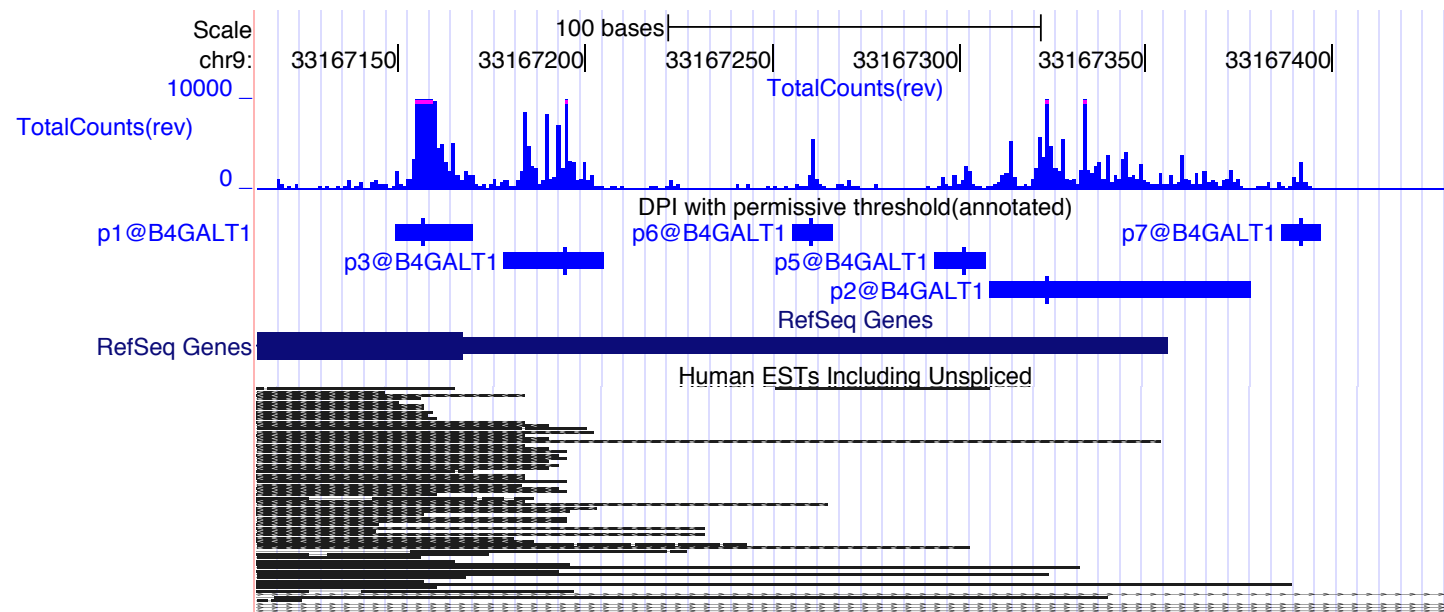

Supplement: Additional file 21: — CAGE peaks and their annotation. Examples of CAGE peaks identified in FANTOM5. Six peaks in the proximal region of B4GALT1 promoters are identified, and their names are indicated as p#@B4GALT1. The track below indicates that all of the peaks are supported by at least one EST (expressed sequence tag) model. [file 13059_2014_560_MOESM21_ESM.pdf]

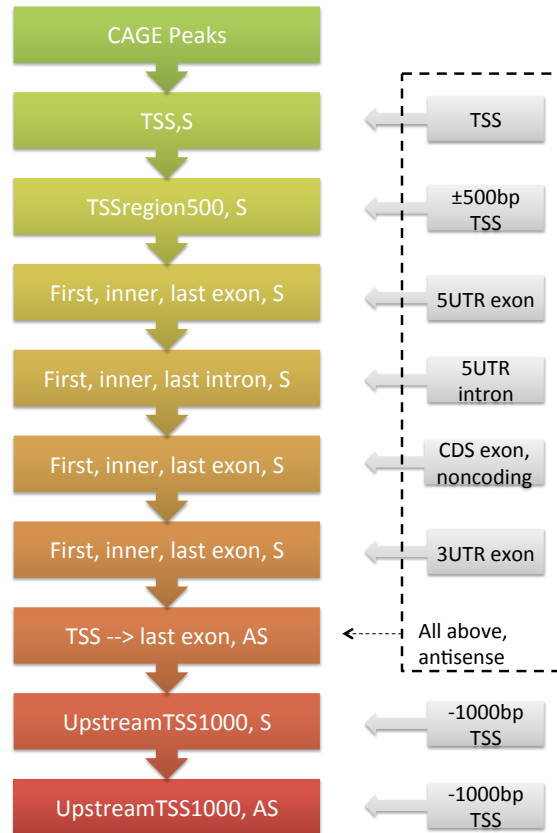

Supplement: Additional file 22: — Classification of CAGE peaks according to the transcript structure. Our hierarchical approach annotates CAGE peaks (left side, colored boxes) with respect to Gencode V10 transcript model structures (right side, grey boxes). The output of one step represents peaks that were not annotated yet and makes the input to the next step, as indicated by the direction of the arrows. The hierarchy is first run for sense transcript models, and then again for anti-sense ones. At the end of the pipeline, peaks are annotated as upstream and downstream (first sense, then antisense) of a TSS. [file 13059_2014_560_MOESM22_ESM.pdf]

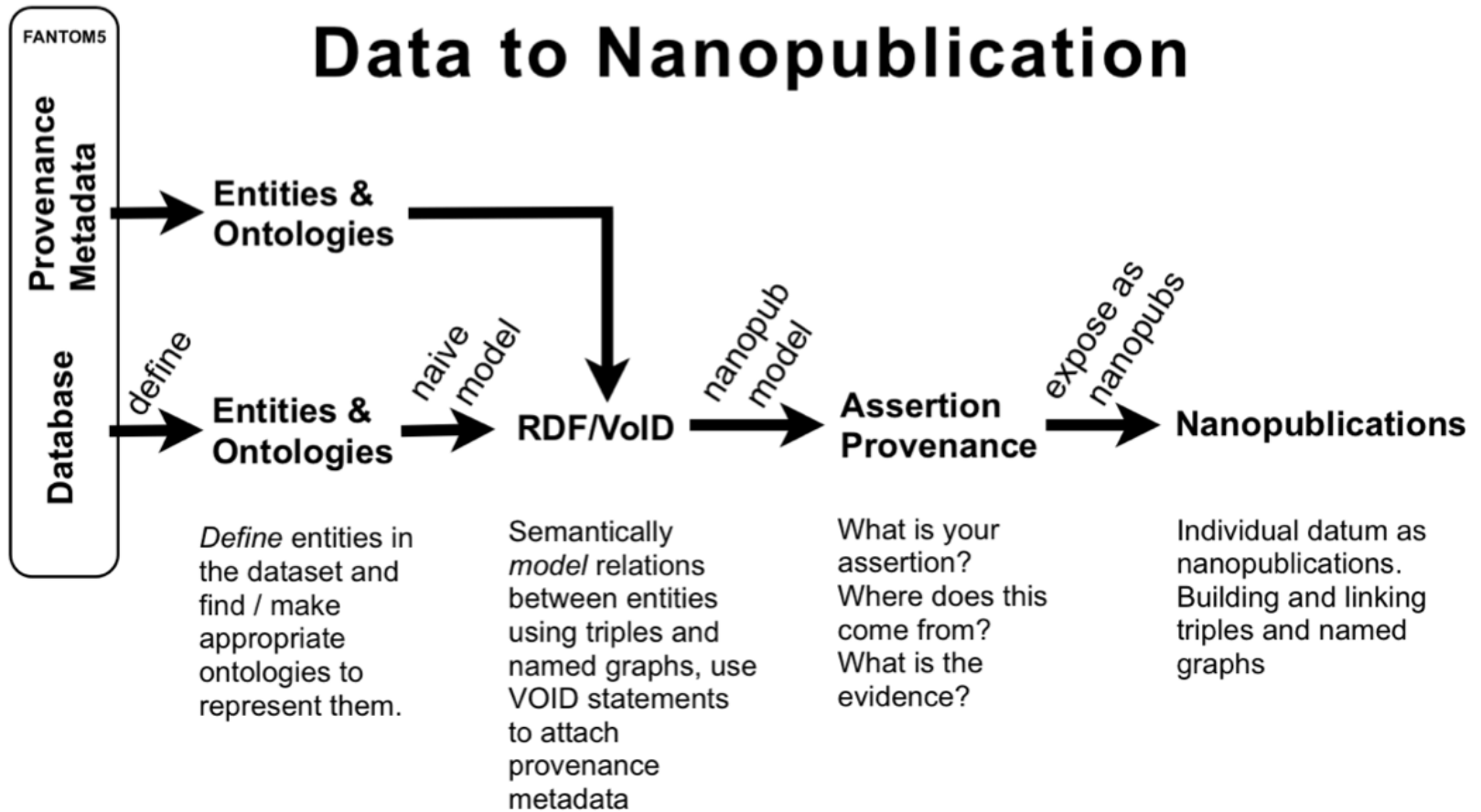

Supplement: Additional file 23: — Workflow converting FANTOM5 data into nanopublications. [file 13059_2014_560_MOESM23_ESM.pdf]
