# Supplementary material for: Gateways to the FANTOM5 promoter level mammalian expression atlas
Source: Genome Biol. 2015 Jan 5;16(1):22. doi: 10.1186/s13059-014-0560-6 (PMC4310165; doi:10.1186/s13059-014-0560-6)
Supplement: Additional file 6: — Information and analysis results on a monocyte profile. The information collected on the samples, like detailed sample and RNA information, highly expressed transcription factors, significant de novo motifs, co-expressed sample clusters, and highly expressed repeats are summarized into a single SSTAR page. [file 13059_2014_560_MOESM6_ESM.pdf]

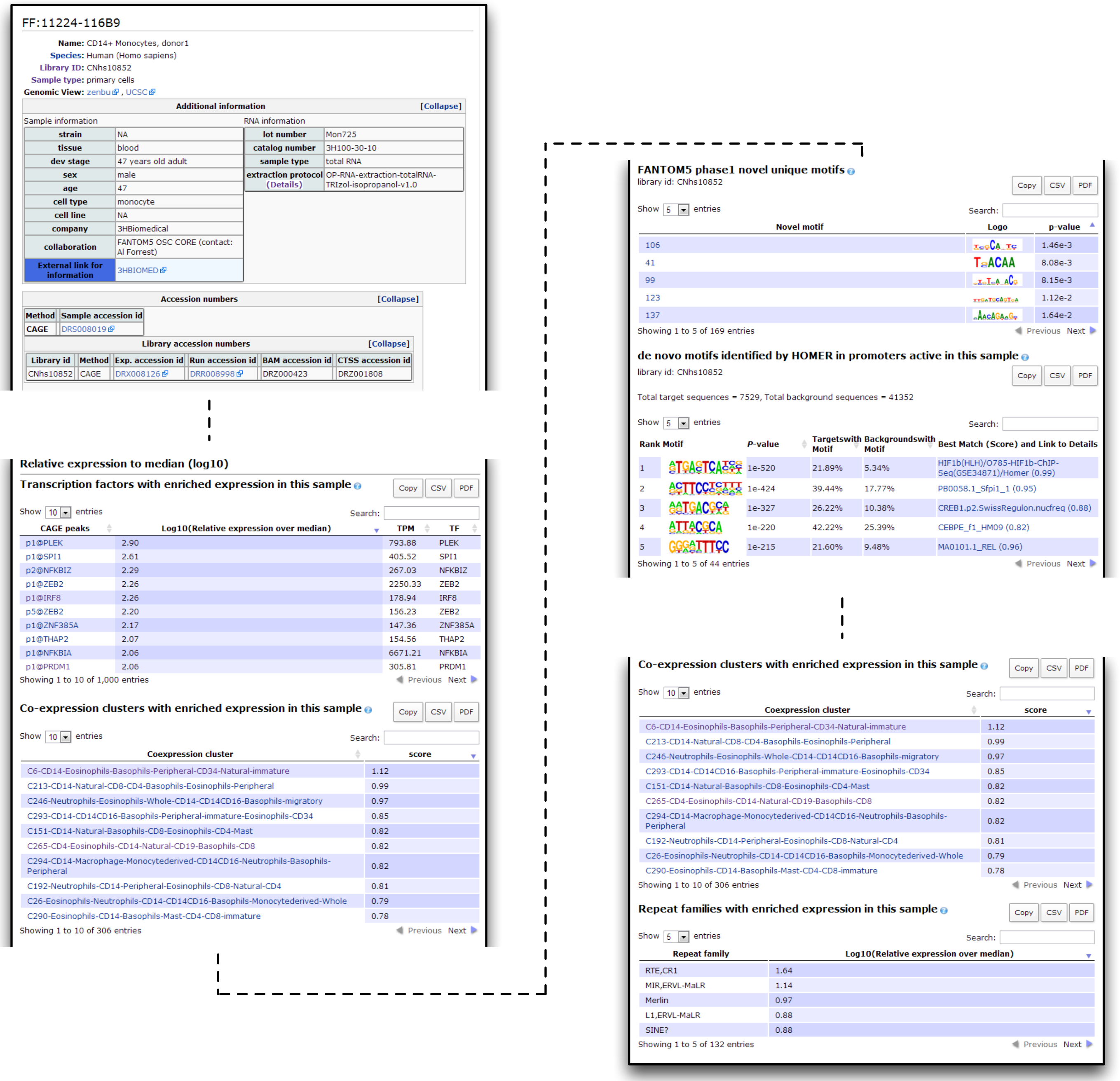

FF:11224-116B9

**Name:** CD14+ Monocytes, donor1
**Species:** Human (Homo sapiens)
**Library ID:** CNhs10852
**Sample type:** primary cells
**Genomic View:** zenbu , UCSC

**Additional information** [Collapse]

Sample information

| | |
|---|---|
| strain | NA |
| tissue | blood |
| dev stage | 47 years old adult |
| sex | male |
| age | 47 |
| cell type | monocyte |
| cell line | NA |
| company | 3HBiomedical |
| collaboration | FANTOM5 OSC CORE (contact: Al Forrest) |
| External link for information | 3HBIOMED |

RNA information

| | |
|---|---|
| lot number | Mon725 |
| catalog number | 3H100-30-10 |
| sample type | total RNA |
| extraction protocol (Details) | OP-RNA-extraction-totalRNA-TRIzol-isopropanol-v1.0 |

**Accession numbers** [Collapse]

| Method | Sample accession id |
|---|---|
| CAGE | DRS008019 |

**Library accession numbers** [Collapse]

| Library id | Method | Exp. accession id | Run accession id | BAM accession id | CTSS accession id |
|---|---|---|---|---|---|
| CNhs10852 | CAGE | DRX008126 | DRR008998 | DRZ000423 | DRZ001808 |

**Relative expression to median (log10)**

**Transcription factors with enriched expression in this sample**

Copy CSV PDF

Show 10 entries Search:

| CAGE peaks | Log10(Relative expression over median) | TPM | TF |
|---|---|---|---|
| p1@PLEK | 2.90 | 793.88 | PLEK |
| p1@SPI1 | 2.61 | 405.52 | SPI1 |
| p2@NFKBIZ | 2.29 | 267.03 | NFKBIZ |
| p1@ZEB2 | 2.26 | 2250.33 | ZEB2 |
| p1@IRF8 | 2.26 | 178.94 | IRF8 |
| p5@ZEB2 | 2.20 | 156.23 | ZEB2 |
| p1@ZNF385A | 2.17 | 147.36 | ZNF385A |
| p1@THAP2 | 2.07 | 154.56 | THAP2 |
| p1@NFKBIA | 2.06 | 6671.21 | NFKBIA |
| p1@PRDM1 | 2.06 | 305.81 | PRDM1 |

Showing 1 to 10 of 1,000 entries Previous Next

**Co-expression clusters with enriched expression in this sample**

Copy CSV PDF

Show 10 entries Search:

| Coexpression cluster | score |
|---|---|
| C6-CD14-Eosinophils-Basophils-Peripheral-CD34-Natural-immature | 1.12 |
| C213-CD14-Natural-CD8-CD4-Basophils-Eosinophils-Peripheral | 0.99 |
| C246-Neutrophils-Eosinophils-Whole-CD14-CD14CD16-Basophils-migratory | 0.97 |
| C293-CD14-CD14CD16-Basophils-Peripheral-immature-Eosinophils-CD34 | 0.85 |
| C151-CD14-Natural-Basophils-CD8-Eosinophils-CD4-Mast | 0.82 |
| C265-CD4-Eosinophils-CD14-Natural-CD19-Basophils-CD8 | 0.82 |
| C294-CD14-Macrophage-Monocytederived-CD14CD16-Neutrophils-Basophils-Peripheral | 0.82 |
| C192-Neutrophils-CD14-Peripheral-Eosinophils-CD8-Natural-CD4 | 0.81 |
| C26-Eosinophils-Neutrophils-CD14-CD14CD16-Basophils-Monocytederived-Whole | 0.79 |
| C290-Eosinophils-CD14-Basophils-Mast-CD4-CD8-immature | 0.78 |

Showing 1 to 10 of 306 entries Previous Next

**FANTOM5 phase1 novel unique motifs**
library id: CNhs10852

Copy CSV PDF

Show 5 entries Search:

| Novel motif | Logo | p-value |
|---|---|---|
| 106 | | 1.46e-3 |
| 41 | | 8.08e-3 |
| 99 | | 8.15e-3 |
| 123 | | 1.12e-2 |
| 137 | | 1.64e-2 |

Showing 1 to 5 of 169 entries Previous Next

**de novo motifs identified by HOMER in promoters active in this sample**
library id: CNhs10852

Copy CSV PDF

Total target sequences = 7529, Total background sequences = 41352

Show 5 entries Search:

| Rank | Motif | *P*-value | Targetswith Motif | Backgroundswith Motif | Best Match (Score) and Link to Details |
|---|---|---|---|---|---|
| 1 | | 1e-520 | 21.89% | 5.34% | HIF1b(HLH)/O785-HIF1b-ChIP-Seq(GSE34871)/Homer (0.99) |
| 2 | | 1e-424 | 39.44% | 17.77% | PB0058.1_Sfpi1_1 (0.95) |
| 3 | | 1e-327 | 26.22% | 10.38% | CREB1.p2.SwissRegulon.nucfreq (0.88) |
| 4 | | 1e-220 | 42.22% | 25.39% | CEBPE_f1_HM09 (0.82) |
| 5 | | 1e-215 | 21.60% | 9.48% | MA0101.1_REL (0.96) |

Showing 1 to 5 of 44 entries Previous Next


**Co-expression clusters with enriched expression in this sample**

Copy CSV PDF

Show 10 entries Search:

| Coexpression cluster | score |
|---|---|
| C6-CD14-Eosinophils-Basophils-Peripheral-CD34-Natural-immature | 1.12 |
| C213-CD14-Natural-CD8-CD4-Basophils-Eosinophils-Peripheral | 0.99 |
| C246-Neutrophils-Eosinophils-Whole-CD14-CD14CD16-Basophils-migratory | 0.97 |
| C293-CD14-CD14CD16-Basophils-Peripheral-immature-Eosinophils-CD34 | 0.85 |
| C151-CD14-Natural-Basophils-CD8-Eosinophils-CD4-Mast | 0.82 |
| C265-CD4-Eosinophils-CD14-Natural-CD19-Basophils-CD8 | 0.82 |
| C294-CD14-Macrophage-Monocytederived-CD14CD16-Neutrophils-Basophils-Peripheral | 0.82 |
| C192-Neutrophils-CD14-Peripheral-Eosinophils-CD8-Natural-CD4 | 0.81 |
| C26-Eosinophils-Neutrophils-CD14-CD14CD16-Basophils-Monocytederived-Whole | 0.79 |
| C290-Eosinophils-CD14-Basophils-Mast-CD4-CD8-immature | 0.78 |

Showing 1 to 10 of 306 entries Previous Next


**Repeat families with enriched expression in this sample**

Copy CSV PDF

Show 5 entries Search:

| Repeat family | Log10(Relative expression over median) |
|---|---|
| RTE,CR1 | 1.64 |
| MIR,ERVL-MaLR | 1.14 |
| Merlin | 0.97 |
| L1,ERVL-MaLR | 0.88 |
| SINE? | 0.88 |

Showing 1 to 5 of 132 entries Previous Next
