# Supplementary material for: Gateways to the FANTOM5 promoter level mammalian expression atlas
Source: Genome Biol. 2015 Jan 5;16(1):22. doi: 10.1186/s13059-014-0560-6 (PMC4310165; doi:10.1186/s13059-014-0560-6)
Supplement: Additional file 7: — Access to individual samples in SSTAR. Two ways to identify samples of interest in SSTAR: by using a list of sample names (orange arrows); by cell type inspection followed by selection of samples of interest (purple arrows). If the sample of interest has one UBERON term associated with it, a search through tissue types can be performed too (blue arrow). [file 13059_2014_560_MOESM7_ESM.pdf]

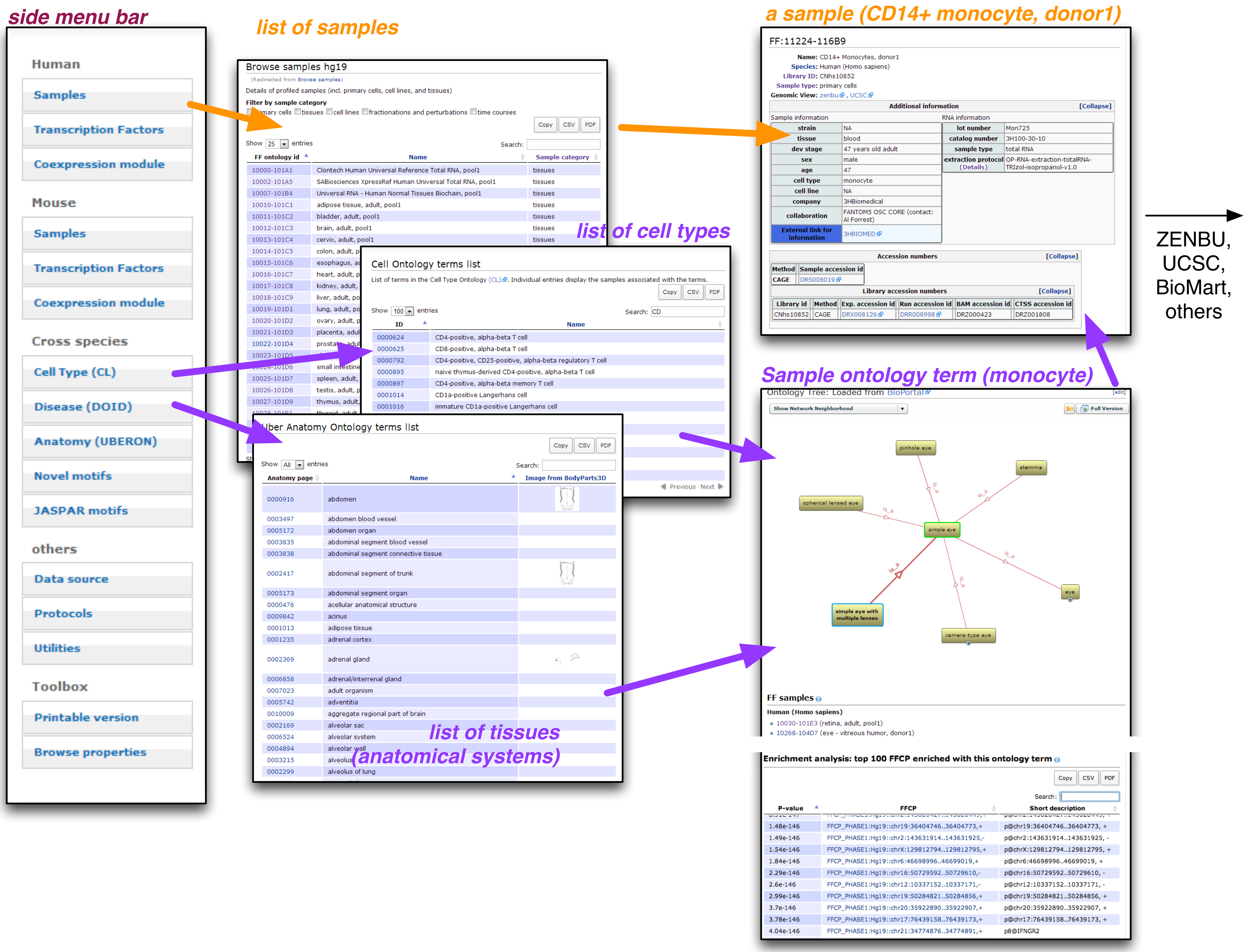
side menu bar
Human
Samples
Transcription Factors
Coexpression module
Mouse
Samples
Transcription Factors
Coexpression module
Cross species
Cell Type (CL)
Disease (DOID)
Anatomy (UBERON)
Novel motifs
JASPAR motifs
others
Data source
Protocols
Utilities
Toolbox
Printable version
Browse properties
list of samples
Browse samples hg19
Details of profiled samples (incl. primary cells, cell lines, and tissues)
Filter by sample category
FF ontology id
Name
Sample category
10000-101A1 Clontech Human Universal Reference Total RNA, pool1 tissues
10002-101A5 SABiosciences XpressRef Human Universal Total RNA, pool1 tissues
10007-101B4 Universal RNA - Human Normal Tissues Biochain, pool1 tissues
10010-101C1 adipose tissue, adult, pool1 tissues
10011-101C2 bladder, adult, pool1 tissues
10012-101C3 brain, adult, pool1 tissues
10013-101C4 cervix, adult, pool1 tissues
list of cell types
Cell Ontology terms list
List of terms in the Cell Type Ontology (CL). Individual entries display the samples associated with the terms.
ID
Name
0000624 CD4-positive, alpha-beta T cell
0000625 CD8-positive, alpha-beta T cell
0000792 CD4-positive, CD25-positive, alpha-beta regulatory T cell
0000895 naive thymus-derived CD4-positive, alpha-beta T cell
0000897 CD4-positive, alpha-beta memory T cell
0001014 CD1a-positive Langerhans cell
0001016 immature CD1a-positive Langerhans cell
Uber Anatomy Ontology terms list
Anatomy page
Name
Image from BodyParts3D
0000916 abdomen
0003497 abdomen blood vessel
0005172 abdomen organ
0003835 abdominal segment blood vessel
0003838 abdominal segment connective tissue
0002417 abdominal segment of trunk
0005173 abdominal segment organ
0000476 acellular anatomical structure
0009842 acinus
0001013 adipose tissue
0001235 adrenal cortex
0002369 adrenal gland
0006858 adrenal/interrenal gland
0007023 adult organism
0005742 adventitia
0010009 aggregate regional part of brain
0002169 alveolar sac
0006524 alveolar system
0004894 alveolar wall
0002299 alveolus of lung
list of tissues
(anatomical systems)
a sample (CD14+ monocyte, donor1)
FF:11224-116B9
Name: CD14+ Monocytes, donor1
Species: Human (Homo sapiens)
Library ID: CNhs10852
Sample type: primary cells
Genomic View: zenbu, UCSC
Additional information
strain NA
tissue blood
dev stage 47 years old adult
sex male
age 47
cell type monocyte
cell line NA
company 3HBiomedical
collaboration FANTOM5 OSC CORE (contact: Al Forrest)
lot number Mon725
catalog number 3H100-30-10
sample type total RNA
extraction protocol OP-RNA-extraction-totalRNA-TRIzol-isopropanol-v1.0
Accession numbers
CAGE DRS008019
Library accession numbers
CNhs10852 CAGE DRX008126 DRR008998 DRZ000423 DRZ001808
ZENBU,
UCSC,
BioMart,
others
Sample ontology term (monocyte)
pinhole eye
stemma
spherical lensed eye
simple eye
eye
simple eye with multiple lenses
camera-type eye
FF samples
Human (Homo sapiens)
10030-101E3 (retina, adult, pool1)
10268-104D7 (eye - vitreous humor, donor1)
Enrichment analysis: top 100 FFCP enriched with this ontology term
P-value
FFCP
Short description
1.48e-146 FFCP_PHASE1:Hg19::chr19:36404746..36404773,+ p@chr19:36404746..36404773, +
1.49e-146 FFCP_PHASE1:Hg19::chr2:143631914..143631925,- p@chr2:143631914..143631925, -
1.54e-146 FFCP_PHASE1:Hg19::chrX:129812794..129812795,+ p@chrX:129812794..129812795, +
1.84e-146 FFCP_PHASE1:Hg19::chr6:46698996..46699019,+ p@chr6:46698996..46699019, +
2.29e-146 FFCP_PHASE1:Hg19::chr16:50729592..50729610,- p@chr16:50729592..50729610, -
2.6e-146 FFCP_PHASE1:Hg19::chr12:10337152..10337171,- p@chr12:10337152..10337171, -
2.99e-146 FFCP_PHASE1:Hg19::chr19:50284821..50284856,+ p@chr19:50284821..50284856, +
3.7e-146 FFCP_PHASE1:Hg19::chr20:35922890..35922907,+ p@chr20:35922890..35922907, +
3.78e-146 FFCP_PHASE1:Hg19::chr17:76439158..76439173,+ p@chr17:76439158..76439173, +
4.04e-146 FFCP_PHASE1:Hg19::chr21:34774876..34774891,+ p8@IFNGR2
