# Supplementary material for: Gateways to the FANTOM5 promoter level mammalian expression atlas
Source: Genome Biol. 2015 Jan 5;16(1):22. doi: 10.1186/s13059-014-0560-6 (PMC4310165; doi:10.1186/s13059-014-0560-6)
Supplement: Additional file 8: — Sample ontology enrichment analysis connected to CAGE peak expression. Results of sample ontology enrichment analysis on 'hematopoietic cell' showed one of the SPI1-related CAGE peaks (p6@SPI1) as enriched. A link to the CAGE peak page where its individual expression pattern can be confirmed. [file 13059_2014_560_MOESM8_ESM.pdf]

Enrichment analysis: top 100 FFCP enriched with this ontology term

[edit]

Copy | CSV | TSV | PDF

Search:

P-value	FFCP	Short description
6.73e-89	FFCP_PHASE1:Hg19::chr16:29757272..29757347,-	p1@C16orf54
7.52e-89	FFCP_PHASE1:Hg19::chr3:107843890..107843896,+	p@chr3:107843890..107843896, +
7.87e-89	FFCP_PHASE1:Hg19::chr7:37382360..37382399,-	p3@ELMO1
1.0e-88	FFCP_PHASE1:Hg19::chr9:95726554..95726616,+	p1@FGD3
1.09e-88	FFCP_PHASE1:Hg19::chr12:7060440..7060453,+	p5@PTPN6
1.43e-88	FFCP_PHASE1:Hg19::chr1:111415757..111415779,+	p1@CD53
1.54e-88	FFCP_PHASE1:Hg19::chr1:31206388..31206521,-	p@chr1:312[illegible]
1.61e-88	FFCP_PHASE1:Hg19::chr11:47400045..47400060,-	p6@SPI1
2.35e-88	FFCP_PHASE1:Hg19::chr6:159466012..159466030,-	[illegible]
3.46e-88	FFCP_PHASE1:Hg19::chr17:7239821..7239902,+	p1@ACAP1
5.37e-88	FFCP_PHASE1:Hg19::chr12:118796915..118796943,-	p2@TAOK3
7.55e-88	FFCP_PHASE1:Hg19::chr2:143886877..143886888,+	p5@ARHGAP[illegible]
8.15e-88	FFCP_PHASE1:Hg19::chr2:182322864..182322885,+	p3@ITGA4

CAGE Expression

[edit]

Search:

Sample	p6@SPI1
Neutrophils, donor3.CNhs11905.11390-118C4	92.00
Eosinophils, donor2.CNhs12548.12245-129H4	72.48
Neutrophils, donor1.CNhs10862.11233-116C9	71.90
Eosinophils, donor3.CNhs12549.12246-129H5	69.80
Neutrophils, donor2.CNhs11959.11314-117C9	65.82
CD14+ monocytes - treated with Salmonella, donor1.CNhs13471.11866-125B3	47.59
CD14+ monocytes - treated with Salmonella, donor2.CNhs13485.11876-125C4	45.96
Whole blood (ribopure), donor090612, donation1.CNhs11672.12182-129A4	42.15
CD14+ monocytes - treated with lipopolysaccharide, donor2.CNhs13533.11875-125C3	40.14
CD14+ monocytes - treated with lipopolysaccharide, donor3.CNhs13545.11885-125D4	38.92
CD14+ monocytes - treated with Salmonella, donor3.CNhs13493.11886-125D5	36.75
Basophils, donor1.CNhs12546.12241-129G9	36.19

Showing 1 to 889 of 889 entries
