# Supplementary material for: Gateways to the FANTOM5 promoter level mammalian expression atlas
Source: Genome Biol. 2015 Jan 5;16(1):22. doi: 10.1186/s13059-014-0560-6 (PMC4310165; doi:10.1186/s13059-014-0560-6)
Supplement: Additional file 11: — Find genes by keyword search. Keyword search in the SSTAR top page enables genes to be found. [file 13059_2014_560_MOESM11_ESM.pdf]

Main Page

FANTOM5 SSTAR (Semantic catalog of Samples, Transcription initiation And Regulators) provide a way to explore samples, transcriptional initiations, and regulators analyzed in the FANTOM5 project. If you have any questions or comments, please contact to fantom@gsc.riken.jp

Search genes [ ] search

		Examples	Browse
**Samples**	Details of profiled samples (incl. primary cells, cell lines, and tissues)	Smooth Muscle Cells - Aortic, donor0	human<br>[illegible]
**Sample** [illegible]	Samples profiled in FANTOM5 are systematically classified by defining FANTON5 [illegible]		

RB SearchResults

Search within Gene names

Key Word: spi1 search

EntrezGene

- Tax_id: 9606=Human, 10090=Mouse

	Symbol	Synonyms	Description	Tax id
6688	SPI1	OF<br>PU.1<br>SFPI1<br>SPI-1<br>SPI-A	spleen focus forming virus (SFFV) proviral integration oncogene spi1	9606
20375	Sfpi1	Dis-1<br>Dis1<br>PU.1<br>Sfpi-1<br>Spi-1<br>Spi1<br>Tcfpu1<br>Tfpu.1	SFFV proviral integration 1	10090
20700	Serpina1a	Aat-2<br>Aat2<br>Dom1<br>PI1<br>[illegible]	serine (or cysteine) peptidase inhibitor, clade A, member 1A	10090
