# Supplementary material for: Gateways to the FANTOM5 promoter level mammalian expression atlas
Source: Genome Biol. 2015 Jan 5;16(1):22. doi: 10.1186/s13059-014-0560-6 (PMC4310165; doi:10.1186/s13059-014-0560-6)
Supplement: Additional file 12: — Access to transcription factors and DNA motifs. The side bar menu (top left) provides links to lists of transcription factors and DNA motifs. A gene page for a transcription factor (on the right) shows detailed information, including binding motifs. A DNA motif page (center) provides a list of associated samples (the center window). [file 13059_2014_560_MOESM12_ESM.pdf]

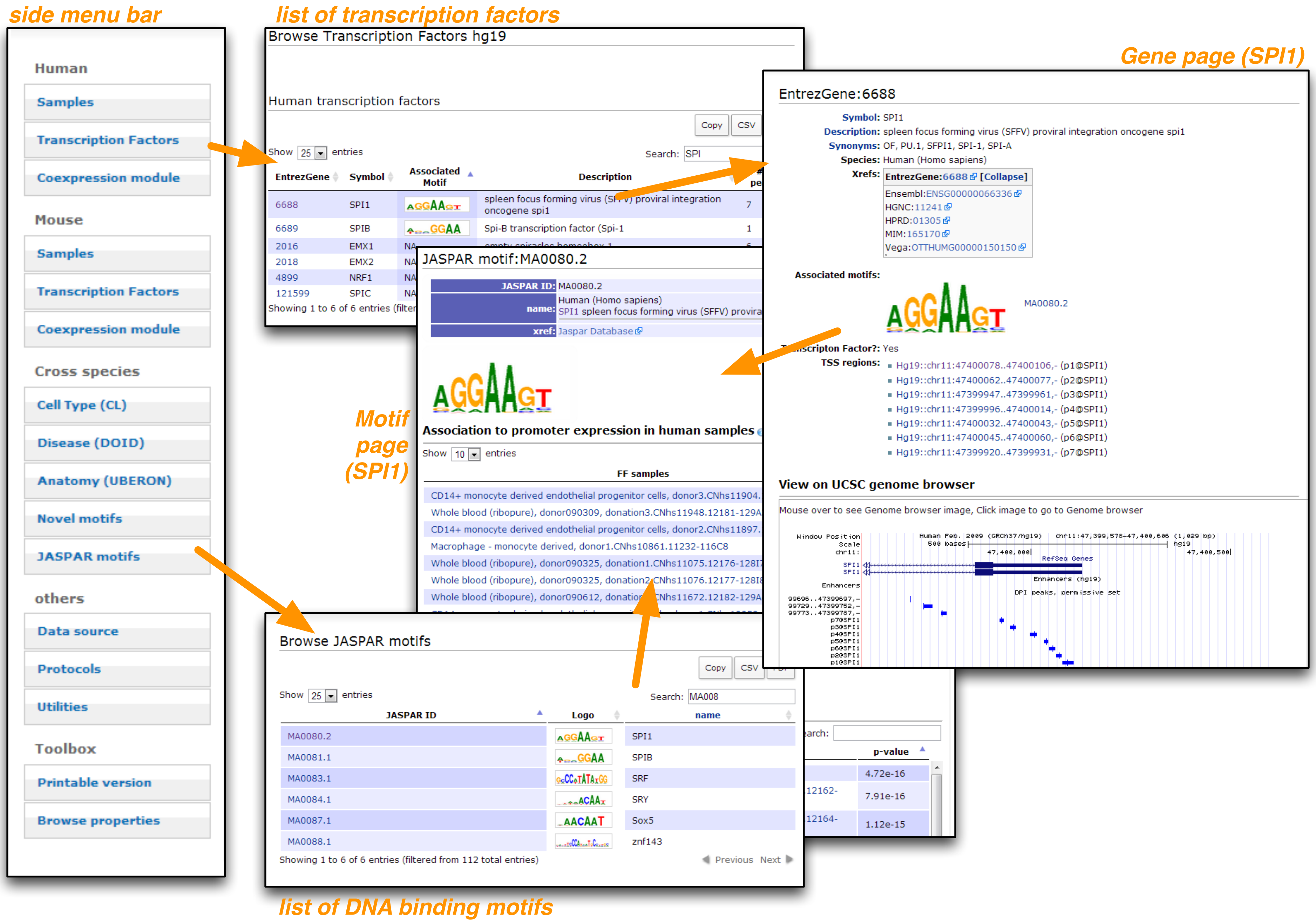

side menu bar
list of transcription factors
Gene page (SPI1)
Motif page (SPI1)
list of DNA binding motifs
Human
Samples
Transcription Factors
Coexpression module
Mouse
Samples
Transcription Factors
Coexpression module
Cross species
Cell Type (CL)
Disease (DOID)
Anatomy (UBERON)
Novel motifs
JASPAR motifs
others
Data source
Protocols
Utilities
Toolbox
Printable version
Browse properties
Browse Transcription Factors hg19
Human transcription factors
Copy
CSV
Show 25 entries
Search: SPI
EntrezGene
Symbol
Associated Motif
Description
6688 SPI1 spleen focus forming virus (SFFV) proviral integration oncogene spi1 7
6689 SPIB Spi-B transcription factor (Spi-1 1
2016 EMX1
2018 EMX2
4899 NRF1
121599 SPIC
Showing 1 to 6 of 6 entries
EntrezGene:6688
Symbol: SPI1
Description: spleen focus forming virus (SFFV) proviral integration oncogene spi1
Synonyms: OF, PU.1, SFPI1, SPI-1, SPI-A
Species: Human (Homo sapiens)
Xrefs: EntrezGene:6688 [Collapse]
Ensembl:ENSG00000066336
HGNC:11241
HPRD:01305
MIM:165170
Vega:OTTHUMG00000150150
Associated motifs:
MA0080.2
Transcripton Factor?: Yes
TSS regions:
Hg19::chr11:47400078..47400106,- (p1@SPI1)
Hg19::chr11:47400062..47400077,- (p2@SPI1)
Hg19::chr11:47399947..47399961,- (p3@SPI1)
Hg19::chr11:47399996..47400014,- (p4@SPI1)
Hg19::chr11:47400032..47400043,- (p5@SPI1)
Hg19::chr11:47400045..47400060,- (p6@SPI1)
Hg19::chr11:47399920..47399931,- (p7@SPI1)
View on UCSC genome browser
Mouse over to see Genome browser image, Click image to go to Genome browser
JASPAR motif:MA0080.2
JASPAR ID: MA0080.2
name: Human (Homo sapiens)
SPI1 spleen focus forming virus (SFFV) proviral
xref: Jaspar Database
Association to promoter expression in human samples
Show 10 entries
FF samples
CD14+ monocyte derived endothelial progenitor cells, donor3.CNhs11904.
Whole blood (ribopure), donor090309, donation3.CNhs11948.12181-129A
CD14+ monocyte derived endothelial progenitor cells, donor2.CNhs11897.
Macrophage - monocyte derived, donor1.CNhs10861.11232-116C8
Whole blood (ribopure), donor090325, donation1.CNhs11075.12176-128I
Whole blood (ribopure), donor090325, donation2.CNhs11076.12177-128I
p-value
4.72e-16
7.91e-16
1.12e-15
Browse JASPAR motifs
Copy
CSV
Show 25 entries
Search: MA008
JASPAR ID
Logo
name
MA0080.2 SPI1
MA0081.1 SPIB
MA0083.1 SRF
MA0084.1 SRY
MA0087.1 Sox5
MA0088.1 znf143
Showing 1 to 6 of 6 entries (filtered from 112 total entries)
Previous Next
