# Supplementary material for: Gateways to the FANTOM5 promoter level mammalian expression atlas
Source: Genome Biol. 2015 Jan 5;16(1):22. doi: 10.1186/s13059-014-0560-6 (PMC4310165; doi:10.1186/s13059-014-0560-6)
Supplement: Additional file 13: — ZENBU Data Explorer. The upper panel shows the data explorer tab and the available options for displaying all data sets (preconfigured views, preconfigured tracks, experiments, annotations). The lower panel is an example of expression experiments where all data sets are listed, including FANTOM5 CAGE. Users can select multiple data sets for individual or pooled graphical representation. [file 13059_2014_560_MOESM13_ESM.pdf]

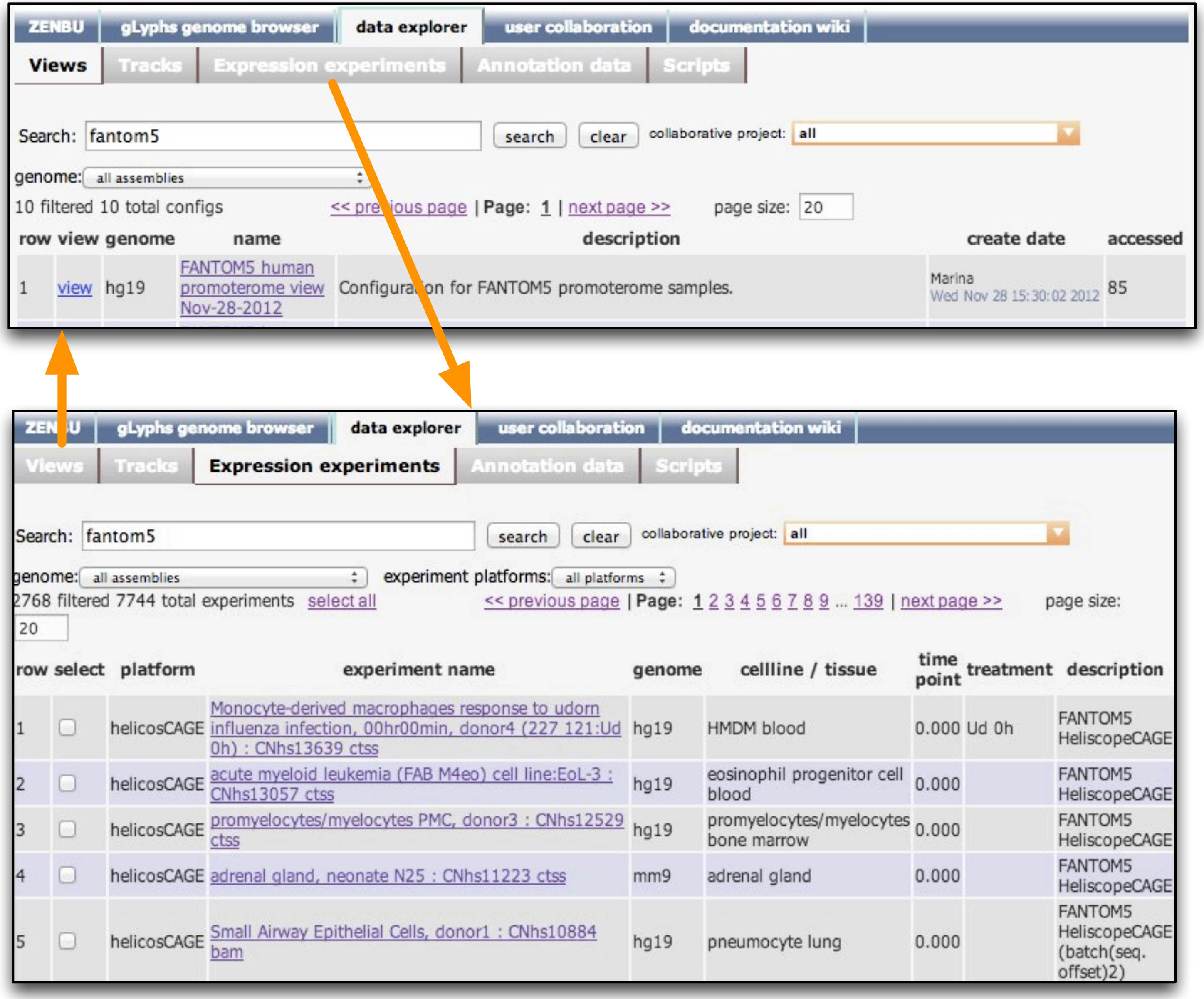

ZENBU
gLyphs genome browser
data explorer
user collaboration
documentation wiki
Views
Tracks
Expression experiments
Annotation data
Scripts
Search: fantom5
search
clear
collaborative project: all
genome: all assemblies
10 filtered 10 total configs
<< previous page | Page: 1 | next page >>
page size: 20
row view genome name description create date accessed
1 view hg19 FANTOM5 human promoterome view Nov-28-2012 Configuration for FANTOM5 promoterome samples. Marina Wed Nov 28 15:30:02 2012 85
ZENBU
gLyphs genome browser
data explorer
user collaboration
documentation wiki
Views
Tracks
Expression experiments
Annotation data
Scripts
Search: fantom5
search
clear
collaborative project: all
genome: all assemblies
experiment platforms: all platforms
2768 filtered 7744 total experiments select all
<< previous page | Page: 1 2 3 4 5 6 7 8 9 ... 139 | next page >>
page size: 20
row select platform experiment name genome cellline / tissue time point treatment description
1 helicosCAGE Monocyte-derived macrophages response to udorn influenza infection, 00hr00min, donor4 (227 121:Ud 0h) : CNhs13639 ctss hg19 HMDM blood 0.000 Ud 0h FANTOM5 HeliscopeCAGE
2 helicosCAGE acute myeloid leukemia (FAB M4eo) cell line:EoL-3 : CNhs13057 ctss hg19 eosinophil progenitor cell blood 0.000 FANTOM5 HeliscopeCAGE
3 helicosCAGE promyelocytes/myelocytes PMC, donor3 : CNhs12529 ctss hg19 promyelocytes/myelocytes bone marrow 0.000 FANTOM5 HeliscopeCAGE
4 helicosCAGE adrenal gland, neonate N25 : CNhs11223 ctss mm9 adrenal gland 0.000 FANTOM5 HeliscopeCAGE
5 helicosCAGE Small Airway Epithelial Cells, donor1 : CNhs10884 bam hg19 pneumocyte lung 0.000 FANTOM5 HeliscopeCAGE (batch(seq. offset)2)
