# Supplementary material for: Gateways to the FANTOM5 promoter level mammalian expression atlas
Source: Genome Biol. 2015 Jan 5;16(1):22. doi: 10.1186/s13059-014-0560-6 (PMC4310165; doi:10.1186/s13059-014-0560-6)
Supplement: Additional file 16: — Annotation export with BioMart. This screenshot shows an example of how to obtain annotations of CAGE peaks, including short descriptions, Human Genome Nomenclature Committee gene IDs, presence of a TATA-box and CpG content. [file 13059_2014_560_MOESM16_ESM.pdf]

FANTOM5
FUNCTIONAL ANNOTATION OF THE MAMMALIAN GENOME

New | Count | Results | URL | XML | Perl | Help

Dataset 8 / 1048124 Entries

hg19 CAGE Peaks (with 12-01-26 annotation)

Filters

Short description (e.g. p1@TP53): %SPI1%

Attributes

CAGE peak ID
Short description
HGNC ID
TATA
CpG

Export all results to [File] [TSV] Unique results only [Go]

Email notification to

View [10] rows as [HTML] Unique results only

CAGE peak ID	Short description	HGNC ID	TATA	CpG
chr11:47399920..47399931,-	p7@SPI1	11241	0	1
chr11:47399947..47399961,-	p3@SPI1	11241	0	1
chr11:47399996..47400014,-	p4@SPI1	11241	0	1
chr11:47400032..47400043,-	p5@SPI1	11241	0	0
chr11:47400045..47400060,-	p6@SPI1	11241	0	0
chr11:47400062..47400077,-	p2@SPI1	11241	0	0
chr11:47400078..47400106,-	p1@SPI1	11241	0	0
chr11:47400277..47400279,-	p8@SPI1	11241	0	0
