# Supplementary material for: Gateways to the FANTOM5 promoter level mammalian expression atlas
Source: Genome Biol. 2015 Jan 5;16(1):22. doi: 10.1186/s13059-014-0560-6 (PMC4310165; doi:10.1186/s13059-014-0560-6)
Supplement: Additional file 17: — Table Extraction Tool. An example of how to export a subset of CAGE peak expression values using TET. Users can select columns in an interactive manner as shown in the left panel, and select rows by specifying the matching string (regular expression). The result can be exported as a table (the right panel) or visualized as a heat map. [file 13059_2014_560_MOESM17_ESM.pdf]

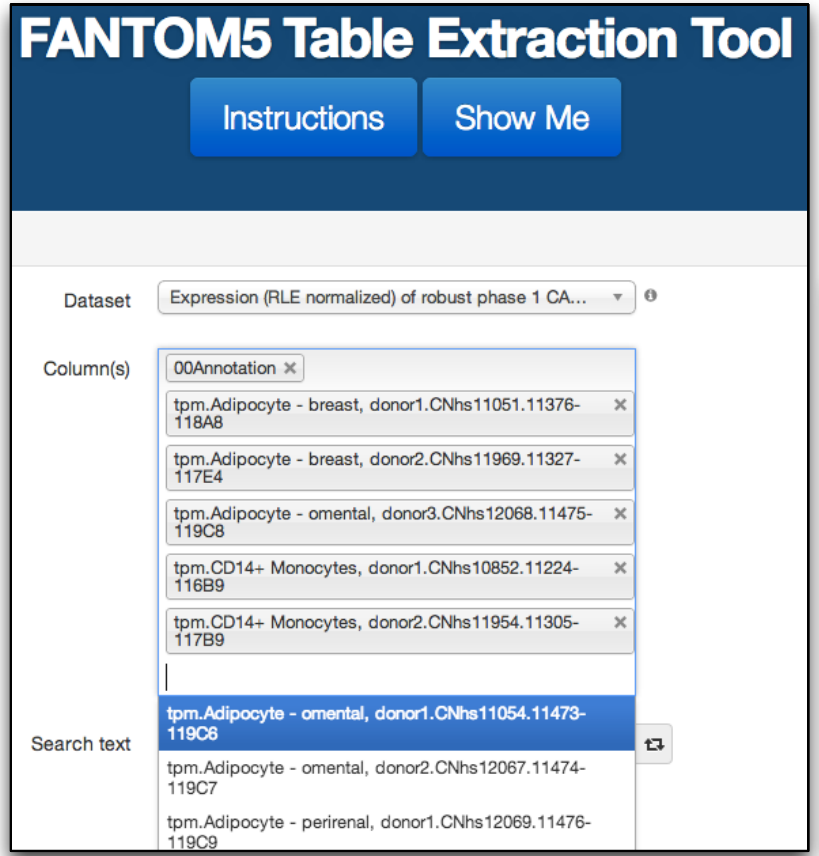

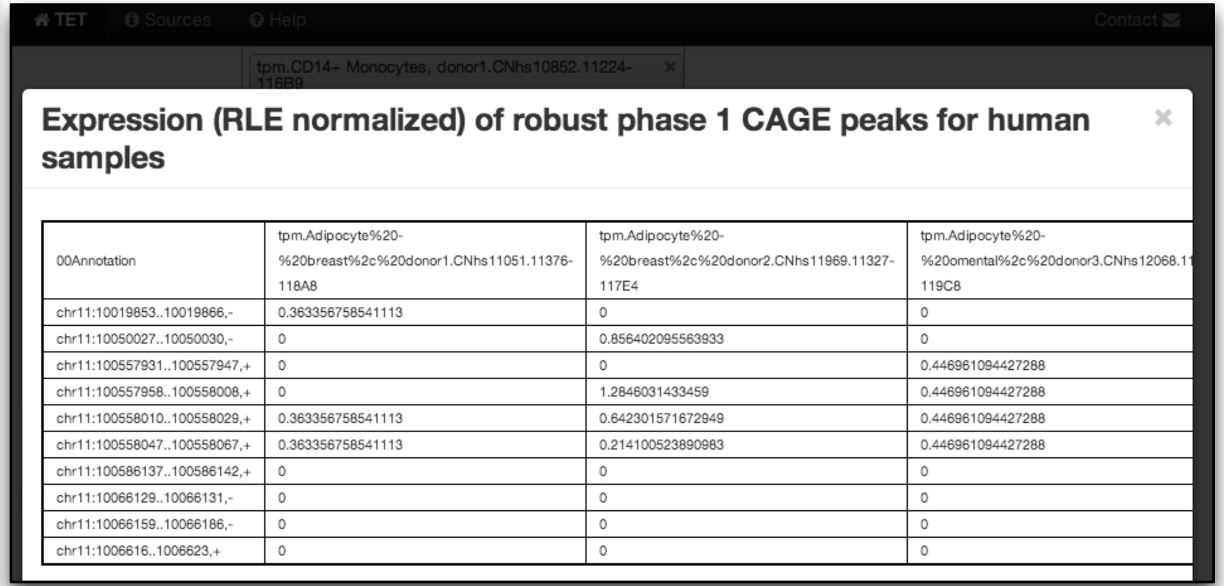

00Annotation	tpm.Adipocyte%20-%20breast%2c%20donor1.CNhs11051.11376-118A8	tpm.Adipocyte%20-%20breast%2c%20donor2.CNhs11969.11327-117E4	tpm.Adipocyte%20-%20omental%2c%20donor3.CNhs12068.11[illegible]119C8
chr11:10019853..10019866,-	0.363356758541113	0	0
chr11:10050027..10050030,-	0	0.856402095563933	0
chr11:100557931..100557947,+	0	0	0.446961094427288
chr11:100557958..100558008,+	0	1.2846031433459	0.446961094427288
chr11:100558010..100558029,+	0.363356758541113	0.642301571672949	0.446961094427288
chr11:100558047..100558067,+	0.363356758541113	0.214100523890983	0.446961094427288
chr11:100586137..100586142,+	0	0	0
chr11:10066129..10066131,-	0	0	0
chr11:10066159..10066186,-	0	0	0
chr11:1006616..1006623,+	0	0	0
