# Supplementary material for: Gateways to the FANTOM5 promoter level mammalian expression atlas
Source: Genome Biol. 2015 Jan 5;16(1):22. doi: 10.1186/s13059-014-0560-6 (PMC4310165; doi:10.1186/s13059-014-0560-6)
Supplement: Additional file 18: — Schema of the annotation pipeline. A nanopublication is a schema built on top of existing semantic web approaches that essentially labels a single scientifically meaningful (publishable) assertion with metadata such that individual assertions are citable and their impact trackable. Nanopublications are composed of three elements: (1) the Assertion; (2) the Provenance metadata of the assertion (for example, authors, methods, funding source, date/time); and (3) the Provenance metadata about the nanopublication itself, in this case called Publication Info. [file 13059_2014_560_MOESM18_ESM.pdf]

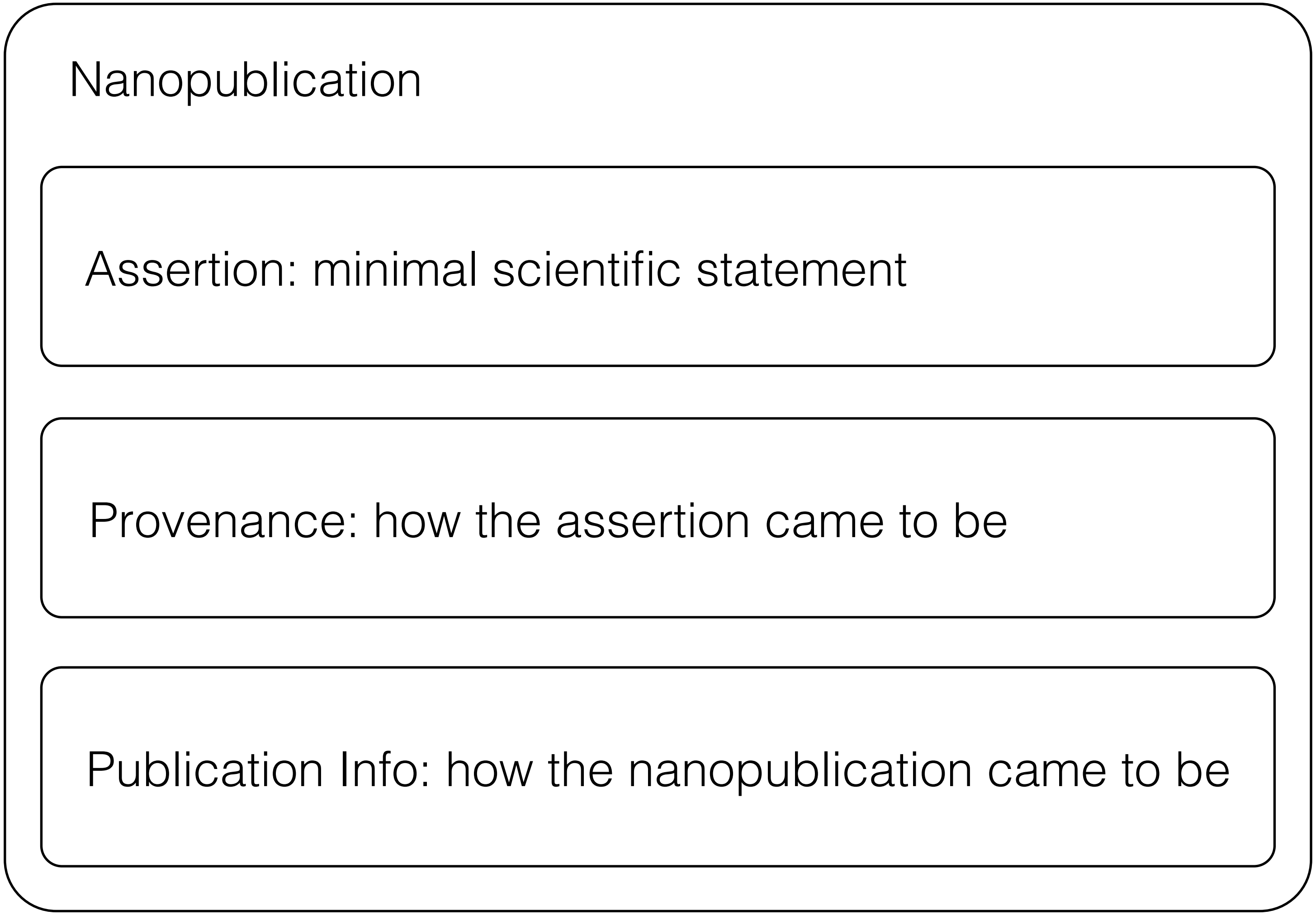
Nanopublication
Assertion: minimal scientific statement
Provenance: how the assertion came to be
Publication Info: how the nanopublication came to be
