# Supplementary material for: Gateways to the FANTOM5 promoter level mammalian expression atlas
Source: Genome Biol. 2015 Jan 5;16(1):22. doi: 10.1186/s13059-014-0560-6 (PMC4310165; doi:10.1186/s13059-014-0560-6)
Supplement: Additional file 19: — An example of a SPARQL query. A SPARQL query that integrates data from three different Linked Data resources: the FANTOM5 nanopublication repository, the FANTOM5 Ontology and Linked Life Data. (A) The variables in the query linking the different datasets together. First the FANTOM5 ontology is queried to find samples from skeletal muscle. Then Linked Life Data is used to link the given gene symbol MYOD to a Bio2RDF resource URL. This Bio2RDF URL is used in the type II nanopublications to identify the CAGE peaks, which are a TSS region for the given gene. Using the type 3 nanopublications, we restrict the search for TSSs to the previously identified sample types that have a tags per million value larger than 0 (meaning that there is evidence for transcription on that region). Finally, the type I nanopublications provide the start and end coordinates for the TSSs. (B) The actual query. [file 13059_2014_560_MOESM19_ESM.pdf]

(A)

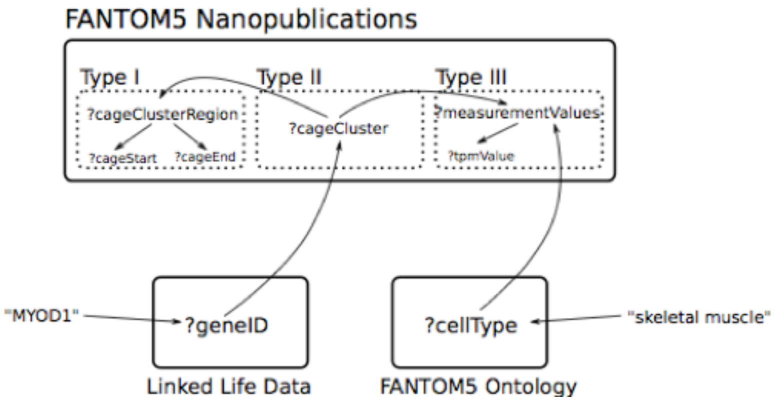

(B)

```
PREFIX np: <http://www.nanopub.org/nschema#>
PREFIX rso: <http://rdf.biosemantics.org/ontologies/referencesequence#>
PREFIX so: <http://purl.org/obo/owl/SO#>
PREFIX ff: <http://purl.obolibrary.org/obo/>
PREFIX entGene: <http://linkedlifedata.com/resource/entrezgene/>
PREFIX lldRel: <http://linkedlifedata.com/resource/relationontology/>
PREFIX uniprot: <http://purl.uniprot.org/taxonomy/>

select ?cageStart ?cageEnd ?cageChromosome ?cellTypeLabel ?tpmValue  where {

# FANTOM5 Ontology
SERVICE <http://agraph.biosemantics.org/catalogs/rajatest/repositories/fantom5-ontology> {
  # CL_0000188 = skeletal muscle cell
  ?cellType rdfs:subClassOf*ff:CL_0000188.
}

# Linked Life Data
{
  ?geneResource entGene:geneSymbol|entrezgene:synonym "MYOD";
    lldRel:expressedInOrganism uniprot:9606 ;
    entGene:hasGeneId ?geneID .
}

# Convert ?geneID to Bio2RDF gene resource URI
BIND (URI(CONCAT("http://bio2rdf.org/geneid:",?geneID)) AS ?bio2rdfGeneID)

# FANTOM5 Nanopublications
SERVICE <http://fantom5.nanopub.org/fantom5/sparql> {

  graph ?rikenAssertion2 { # Type 2 Nanopublicatons
    ?cageCluster rso:is_observation_of ?tss.
    ?tss so:part_of ?bio2rdfGeneID.
  }

  graph ?rikenAssertion1 { # Type 1 Nanopublicatons
    ?cageCluster rso:mapsTo ?cageClusterRegion.
    ?cageClusterRegion  rso:start ?cageStart;
                        rso:end ?cageEnd;
                        rso:regionOf ?cageChromosome.
  }

  graph ?rikenAssertion3 { # Type 3 Nanopublicatons
    ?cageCluster so:associated_with ?measurementValues;
                 rso:observed_in ?cellType.
    ?measurementValues ff:IAO_0000004 ?tpmValue.

    FILTER (?tpmValue >= 0)
  }
}
} order by desc (?cageStart)
~
```
