# Supplementary material for: Gateways to the FANTOM5 promoter level mammalian expression atlas
Source: Genome Biol. 2015 Jan 5;16(1):22. doi: 10.1186/s13059-014-0560-6 (PMC4310165; doi:10.1186/s13059-014-0560-6)
Supplement: Additional file 20: — An example of a SPARQL query. Retrieved data for the query in Additional file 19 is shown. [file 13059_2014_560_MOESM20_ESM.pdf]

cageStart	cageEnd	cageChromosome	cellTypeLabel	tpmValue
17741254	17741282	http://rdf.biosemantics.org/data/genomeassemblies/hg19#chr11	skeletal muscle, fetal, donor1	22.0365
17741254	17741282	http://rdf.biosemantics.org/data/genomeassemblies/hg19#chr11	Skeletal Muscle Satellite Cells, donor3	3.40907
17741254	17741282	http://rdf.biosemantics.org/data/genomeassemblies/hg19#chr11	Skeletal Muscle Cells, donor4	0.462146
17741254	17741282	http://rdf.biosemantics.org/data/genomeassemblies/hg19#chr11	Skeletal Muscle Cells, donor5	2.14905
17741254	17741282	http://rdf.biosemantics.org/data/genomeassemblies/hg19#chr11	Skeletal Muscle Cells, donor6	4.1351
17741254	17741282	http://rdf.biosemantics.org/data/genomeassemblies/hg19#chr11	Skeletal Muscle Cells, donor1	34.8981
17741254	17741282	http://rdf.biosemantics.org/data/genomeassemblies/hg19#chr11	Skeletal muscle cells differentiated into Myotubes - multinucleated, donor1	11.8413
17741254	17741282	http://rdf.biosemantics.org/data/genomeassemblies/hg19#chr11	Skeletal Muscle Satellite Cells, donor1	0.322261
17741254	17741282	http://rdf.biosemantics.org/data/genomeassemblies/hg19#chr11	skeletal muscle, adult, pool1	1.43728
17741254	17741282	http://rdf.biosemantics.org/data/genomeassemblies/hg19#chr11	Skeletal Muscle Satellite Cells, donor2	0.725083
17741254	17741282	http://rdf.biosemantics.org/data/genomeassemblies/hg19#chr11	skeletal muscle - soleus muscle, donor1	1.29486
17741228	17741243	http://rdf.biosemantics.org/data/genomeassemblies/hg19#chr11	skeletal muscle, fetal, donor1	3.47944
17741228	17741243	http://rdf.biosemantics.org/data/genomeassemblies/hg19#chr11	Skeletal Muscle Satellite Cells, donor3	0.681813
17741228	17741243	http://rdf.biosemantics.org/data/genomeassemblies/hg19#chr11	Skeletal Muscle Cells, donor4	0.308097
17741228	17741243	http://rdf.biosemantics.org/data/genomeassemblies/hg19#chr11	Skeletal Muscle Cells, donor5	0.460511
17741228	17741243	http://rdf.biosemantics.org/data/genomeassemblies/hg19#chr11	Skeletal Muscle Cells, donor6	0.689183
17741228	17741243	http://rdf.biosemantics.org/data/genomeassemblies/hg19#chr11	Skeletal Muscle Cells, donor1	2.90012
17741228	17741243	http://rdf.biosemantics.org/data/genomeassemblies/hg19#chr11	Skeletal muscle cells differentiated into Myotubes - multinucleated, donor1	0.89032
17741228	17741243	http://rdf.biosemantics.org/data/genomeassemblies/hg19#chr11	Skeletal Muscle Satellite Cells, donor1	0
17741228	17741243	http://rdf.biosemantics.org/data/genomeassemblies/hg19#chr11	skeletal muscle, adult, pool1	0.102663
17741228	17741243	http://rdf.biosemantics.org/data/genomeassemblies/hg19#chr11	Skeletal Muscle Satellite Cells, donor2	0.145017
17741228	17741243	http://rdf.biosemantics.org/data/genomeassemblies/hg19#chr11	skeletal muscle - soleus muscle, donor1	0.647429
17741206	17741215	http://rdf.biosemantics.org/data/genomeassemblies/hg19#chr11	skeletal muscle, fetal, donor1	6.95889
17741206	17741215	http://rdf.biosemantics.org/data/genomeassemblies/hg19#chr11	Skeletal Muscle Satellite Cells, donor3	0.25568
17741206	17741215	http://rdf.biosemantics.org/data/genomeassemblies/hg19#chr11	Skeletal Muscle Cells, donor4	0.308097
17741206	17741215	http://rdf.biosemantics.org/data/genomeassemblies/hg19#chr11	Skeletal Muscle Cells, donor5	0.460511
17741206	17741215	http://rdf.biosemantics.org/data/genomeassemblies/hg19#chr11	Skeletal Muscle Cells, donor6	0.137837
17741206	17741215	http://rdf.biosemantics.org/data/genomeassemblies/hg19#chr11	Skeletal Muscle Cells, donor1	2.70678
17741206	17741215	http://rdf.biosemantics.org/data/genomeassemblies/hg19#chr11	Skeletal muscle cells differentiated into Myotubes - multinucleated, donor1	0.801288
17741206	17741215	http://rdf.biosemantics.org/data/genomeassemblies/hg19#chr11	Skeletal Muscle Satellite Cells, donor1	0
17741206	17741215	http://rdf.biosemantics.org/data/genomeassemblies/hg19#chr11	skeletal muscle, adult, pool1	0.102663
17741206	17741215	http://rdf.biosemantics.org/data/genomeassemblies/hg19#chr11	Skeletal Muscle Satellite Cells, donor2	0
17741206	17741215	http://rdf.biosemantics.org/data/genomeassemblies/hg19#chr11	skeletal muscle - soleus muscle, donor1	0.647429
17741111	17741124	http://rdf.biosemantics.org/data/genomeassemblies/hg19#chr11	skeletal muscle, fetal, donor1	8.1187
17741111	17741124	http://rdf.biosemantics.org/data/genomeassemblies/hg19#chr11	Skeletal Muscle Satellite Cells, donor3	16.7896
17741111	17741124	http://rdf.biosemantics.org/data/genomeassemblies/hg19#chr11	Skeletal Muscle Cells, donor4	8.47267
17741111	17741124	http://rdf.biosemantics.org/data/genomeassemblies/hg19#chr11	Skeletal Muscle Cells, donor5	12.4338
17741111	17741124	http://rdf.biosemantics.org/data/genomeassemblies/hg19#chr11	Skeletal Muscle Cells, donor6	11.1648
17741111	17741124	http://rdf.biosemantics.org/data/genomeassemblies/hg19#chr11	Skeletal Muscle Cells, donor1	162.793
17741111	17741124	http://rdf.biosemantics.org/data/genomeassemblies/hg19#chr11	Skeletal muscle cells differentiated into Myotubes - multinucleated, donor1	36.5031
17741111	17741124	http://rdf.biosemantics.org/data/genomeassemblies/hg19#chr11	Skeletal Muscle Satellite Cells, donor1	0.966782
17741111	17741124	http://rdf.biosemantics.org/data/genomeassemblies/hg19#chr11	skeletal muscle, adult, pool1	15.9127
17741111	17741124	http://rdf.biosemantics.org/data/genomeassemblies/hg19#chr11	Skeletal Muscle Satellite Cells, donor2	3.4804
17741111	17741124	http://rdf.biosemantics.org/data/genomeassemblies/hg19#chr11	skeletal muscle - soleus muscle, donor1	1.29486
17741084	17741094	http://rdf.biosemantics.org/data/genomeassemblies/hg19#chr11	skeletal muscle, fetal, donor1	2.31963
17741084	17741094	http://rdf.biosemantics.org/data/genomeassemblies/hg19#chr11	Skeletal Muscle Satellite Cells, donor3	2.38635
17741084	17741094	http://rdf.biosemantics.org/data/genomeassemblies/hg19#chr11	Skeletal Muscle Cells, donor4	0.462146
17741084	17741094	http://rdf.biosemantics.org/data/genomeassemblies/hg19#chr11	Skeletal Muscle Cells, donor5	1.53504
17741084	17741094	http://rdf.biosemantics.org/data/genomeassemblies/hg19#chr11	Skeletal Muscle Cells, donor6	1.92971
17741084	17741094	http://rdf.biosemantics.org/data/genomeassemblies/hg19#chr11	Skeletal Muscle Cells, donor1	8.41035
17741084	17741094	http://rdf.biosemantics.org/data/genomeassemblies/hg19#chr11	Skeletal muscle cells differentiated into Myotubes - multinucleated, donor1	5.07483
17741084	17741094	http://rdf.biosemantics.org/data/genomeassemblies/hg19#chr11	Skeletal Muscle Satellite Cells, donor1	0.322261
17741084	17741094	http://rdf.biosemantics.org/data/genomeassemblies/hg19#chr11	skeletal muscle, adult, pool1	4.82515
17741084	17741094	http://rdf.biosemantics.org/data/genomeassemblies/hg19#chr11	Skeletal Muscle Satellite Cells, donor2	1.01512
17741084	17741094	http://rdf.biosemantics.org/data/genomeassemblies/hg19#chr11	skeletal muscle - soleus muscle, donor1	1.94229
